# The mechanisms of potassium loss in acute myocardial ischemia: New insights from computational simulations

**DOI:** 10.3389/fphys.2023.1074160

**Published:** 2023-02-27

**Authors:** Jose M. Ferrero, Ana Gonzalez-Ascaso, Jose F. Rodriguez Matas

**Affiliations:** ^1^ Centro de Investigacion e Innovacion en Bioingenieria, Universitat Politecnica de Valencia, Valencia, Spain; ^2^ Dipartimento di Chimica, Materiali e Ingegneria Chimica ”Giulio Natta”, Politecnico di Milano, Milan, Italy

**Keywords:** hyperkalemia, myocardial ischemia, injury current, computational model, alternans, potassium loss

## Abstract

Acute myocardial ischemia induces hyperkalemia (accumulation of extracellular potassium), a major perpetrator of lethal reentrant ventricular arrhythmias. Despite considerable experimental efforts to explain this pathology in the last decades, the intimate mechanisms behind hyperkalemia remain partially unknown. In order to investigate these mechanisms, we developed a novel computational model of acute myocardial ischemia which couples a) an electrophysiologically detailed human cardiomyocyte model that incorporates modifications to account for ischemia-induced changes in transmembrane currents, with b) a model of cardiac tissue and extracellular *K* ^+^ transport. The resulting model is able to reproduce and explain the triphasic time course of extracellular *K* ^+^ concentration within the ischemic zone, with values of ${\left[K^{+}\right]}_{o}$ close to 14 mmol/L in the central ischemic zone after 30 min. In addition, the formation of a ${\left[K^{+}\right]}_{o}$ border zone of approximately 1.2 cm 15 min after the onset of ischemia is predicted by the model. Our results indicate that the primary rising phase of ${\left[K^{+}\right]}_{o}$ is mainly due to the imbalance between *K* ^+^ efflux, that increases slightly, and *K* ^+^ influx, that follows a reduction of the NaK pump activity by more than 50%. The onset of the plateau phase is caused by the appearance of electrical alternans (a novel mechanism identified by the model), which cause an abrupt reduction in the *K* ^+^ efflux. After the plateau, the secondary rising phase of ${\left[K^{+}\right]}_{o}$ is caused by a subsequent imbalance between the *K* ^+^ influx, which continues to decrease slowly, and the *K* ^+^ efflux, which remains almost constant. Further, the study shows that the modulation of these mechanisms by the electrotonic coupling is the main responsible for the formation of the ischemic border zone in tissue, with *K* ^+^ transport playing only a minor role. Finally, the results of the model indicate that the injury current established between the healthy and the altered tissue is not sufficient to depolarize non-ischemic cells within the healthy tissue.

## 1 Introduction

Globally, cardiovascular disease (CVD) remains the leading cause of death and disability, accounting for around 18.5 million deaths every year, approximately one-third of all deaths globally (Roth et al., 2020). Among CVD, atherosclerotic coronary artery disease is the most common pathology. This disease may cause a partial or complete occlusion of a coronary artery which, in turn, causes myocardial ischemia and infarction. If ischemia is regional (the most common case) and affects only a part of the myocardium, it introduces abnormal heterogeneity in the tissue. Indeed, resting membrane potential, action potential (AP) duration and effective refractory period, among others, may differ from one site to another. This ischemia-induced heterogeneity provides an important pro-arrhythmic substrate (Kléber, 1984; Janse and Wit, 1989; Coronel, 1994).

The acute phase of myocardial ischemia corresponds to the first 30–60 min after coronary artery occlusion and is associated with a high incidence of arrhythmic events (Smith et al., 1995; Yan et al., 2004; Cascio et al., 2005). During the first 30 min, the period in which this study will focus, ischemia is characterized by important metabolic changes inducing profound electrophysiological alterations in the behavior of affected cardiomyocytes. The main metabolic changes include a reduction in intracellular adenosine triphosphate (ATP), changes alterations in intracellular adenosine diphosphate (ADP), a reduction of tissue pH, and an increase in lysophosphatidylcholine (LPC) (Daleau, 1999; Sakamoto et al., 2000; Terkildsen et al., 2007). These changes cause alterations in the cell resting membrane potential, which becomes less negative, and modulate the inward and outward transmembrane currents during the AP, leading to hyperkalemia (an increase in extracellular potassium concentration, ${\left[K^{+}\right]}_{o}$ ). In the time domain, direct assessment of potassium loss during acute ischemia in different animal models has identified a time course of ${\left[K^{+}\right]}_{o}$ characterized by three distinctive phases (Hill and Gettes, 1980; Coronel et al., 1991; Wilde and Aksnes, 1995). A few seconds after the occlusion, ${\left[K^{+}\right]}_{o}$ begins to rise, reaching a plateau or experiencing a minor decline after approximately 5–10 min after the onset of ischemia. After the plateau phase, a secondary rise in ${\left[K^{+}\right]}_{o}$ occurs. Interestingly, this triphasic pattern has been observed in different animal models (Hill and Gettes, 1980; Weiss and Shine, 1982a; Coronel et al., 1988; Wilde and Aksnes, 1995) under different stimulation frequencies (Harper et al., 1993; Wilde and Aksnes, 1995). In the spatial domain, a central ischemic zone (CIZ), within which cells exhibit the highest degree of hyperkalemia, dynamically develops. The CIZ is surrounded by an ischemic border zone (BZ) of around 1 cm, within which cells are partially affected by hyperkalemia, which declines towards the myocardial normal zone (Hill and Gettes, 1980; Coronel et al., 1988; Coronel et al., 1991).

The increase in extracellular potassium is related to alterations of the electrical behavior of ischemic cardiomyocytes (Janse and Wit, 1989). Specifically, resting membrane potential increases (which reduces cell excitability), action potential duration shortens and conduction velocity diminishes, among other alterations. These electrophysiological changes provide a potential substrate for the generation of arrhythmic events leading to ventricular fibrillation and sudden cardiac death (Harris et al., 1954; Smith et al., 1995). The evidence comes from experiments involving isolated whole hearts subject to regional acute myocardial ischemia (Harris et al., 1954; Janse et al., 1980; Janse and Kléber, 1981; Weiss and Shine, 1981; Weiss and Shine, 1982b; Coronel et al., 1989; Janse and Wit, 1989; Coronel, 1994; Weiss et al., 2017). In these experiments, ischemia (and hyperkalemia in particular) was found to promote unidirectional block and reentry due to the partial or complete loss of cell excitability provoked by extracellular potassium accumulation. Also, the appearance of an “injury current” flowing from the ischemic zone to the normal zone has been hypothesised to induce a source-sink imbalance which could induce ectopic activity that would act as a trigger for reentry (Janse et al., 1980; Janse and van Capelle, 1982; Coronel et al., 1991; Coronel, 1994). Computer simulations have also highlighted the arrhythmogenic effects of ischemia-induced hyperkalemia in virtual 2D tissues (Ferrero et al., 2003; Tice et al., 2007; Trénor et al., 2007; Romero et al., 2009) and 3D virtual bi-ventricular heart models (Mena Tobar et al., 2018; Carpio et al., 2022).

Despite the importance of this phenomenon, and the number of experimental studies trying to elucidate the ionic and tissue related mechanisms behind the increase in ${\left[K^{+}\right]}_{o}$ (Hill and Gettes, 1980; Weiss and Shine, 1982a; Kléber, 1984; Coronel et al., 1988; Coronel et al., 1991; Wilde and Aksnes, 1995), the intimate mechanisms responsible for extracellular potassium accumulation are still uncertain (Wilde and Aksnes, 1995; Shivkumar et al., 1997; Weiss et al., 2017). In particular, the relative individual contributions of the different ionic currents to extracellular potassium accumulation has yet to be established. Also, the triphasic nature of the ${\left[K^{+}\right]}_{o}$ time course (Kléber, 1984) remains to be fully explained. Most of the experimental work was conducted from the late 1970s to the early 1990s, and the fact that few new experimental reports have been published since then may be due to the difficulty in designing new experiments to obtain further evidence of the hyperkalemia mechanisms.

Indeed, using solely experimental means to elucidate the intimate mechanisms responsible for ${\left[K^{+}\right]}_{o}$ accumulation has severe limitations, as it is not currently possible to record individual transmembrane ionic currents simultaneously with the APs and ${\left[K^{+}\right]}_{o}$ in cardiac tissue during acute ischemia. For this reason, efforts have been made to use computational simulations using electrophysiologically detailed models of the AP and its underlying transmembrane ionic currents to explore the mechanisms behind this phenomenon (Rodríguez et al., 2002; Potse et al., 2007a; Potse et al., 2007b; Terkildsen et al., 2007; Niederer, 2013). Although these models were able to partially explain ischemia-induced hyperkalemia, they suffered from important limitations that hindered the soundness of the results. In the pioneering work by Rodríguez et al. (2002), the study was only carried out in a single isolated virtual guinea pig cardiomyocyte and was limited to the initial potassium rise and the plateau. In a subsequent study, Terkildsen et al. (2007) also addressed the mechanisms of the secondary potassium rise but, similarly to the former study, it was limited to an isolated cardiomyocyte and did not account for tissue effects. Later, the role of potassium transport in the development of the potassium BZ was studied in a biventricular human heart model (Potse et al., 2007a; Potse et al., 2007b). However, the model assumed a constant net potassium efflux from the cells, thus uncoupling the electrophysiological behavior of the cell from the extracellular potassium accumulation. Finally, the spatio-temporal evolution of ionic concentrations across the ischemic BZ was then studied by Niederer (2013) by means of a model of cardiac tissue accounting for the diffusion of different metabolites coupled with an electrophysiologically detailed model of the AP. However, the model only considers the diastolic currents, neglecting the electrical excitation and propagation within the tissue. Even though the model predicts the potassium efflux and the diffusion of extracellular potassium, the size of the BZ and the maximum ${\left[K^{+}\right]}_{o}$ are much smaller than those reported experimentally (Coronel, 1994).

Understanding the mechanisms that lead to hyperkalemia would enable a better understanding of its arrhythmogenic effects and the development of more solid approaches to its treatment. With this in mind, this work attempts to study, with the aid of computer simulations, the intimate mechanisms underlying the increase in ${\left[K^{+}\right]}_{o}$ during the first 30 min after the onset of ischemia. For this purpose, a novel electrophysiologically detailed model of the ischemic cell and tissue was developed. The cellular model is based on the O’Hara model of the human ventricular cardiac AP (O’Hara et al., 2011), modified to simulate ischemia by incorporating the effects of ischemia-related metabolites on ionic transmembrane currents. The model also includes an extra equation to account for the dynamic changes in extracellular potassium. The model was then coupled to a tissue model which includes electrophysiology-transport equations to investigate the spatial and temporal dispersion of ${\left[K^{+}\right]}_{o}$ across the ischemic BZ. Ionic concentrations, potassium fluxes across the cell membrane, the transmembrane potential and the extracellular potential were monitored to elucidate the basis of cellular potassium loss during acute myocardial ischemia.

## 2 Materials and methods

### 2.1 Virtual tissue description

In this work, “0D simulations” corresponds to a virtual single isolated ventricular cardiomyocyte, and “1D simulations” to a virtual myocardial one-dimensional strand 4 cm in length. In the latter case, in order to simulate regional ischemia, the first 2 cm correspond to normal cells (“normoxic tissue”), whereas the rest of the fiber was subject to ischemic conditions (“altered tissue”), as described below.

### 2.2 Action potential model

Unless otherwise stated, the simulations were carried out using a modified version of the O’Hara model (O’Hara et al., 2011). This model is widely accepted to simulate APs and the underlying ionic currents in human ventricular cardiomyocytes, and was adopted by an expert group of the FDA as the starting point for developing an *in silico* model suitable for regulatory decision making (Colatsky et al., 2016). The model includes mathematical descriptions of fifteen transmembrane currents flowing through ion channels, pumps and exchangers, as well as the calcium transients involved in the calcium-induced calcium release process, and calcium buffering in the intracellular medium. In particular, the model includes seven different ionic currents that carry potassium ions, namely the rapid delayed rectifier potassium current (*I*_*Kr*_), the slow delayed rectifier potassium current (*I*_*Ks*_), the transient outward potassium current (*I*_*to*_), the inward rectifier potassium current (*I*_*K*1_), the background potassium current (*I*_*Kb*_), the potassium component of the L-type calcium current (*I*_*CaL*_) and the sodium/potassium (NaK) pump (*I*_*NaK*_).

To obtain realistic values of AP upstroke velocity and propagation velocity, the formulation of *I*_*Na*_ and *I*_*NaL*_ were modified as in Carpio et al. (2019) [which, in turn, includes the modifications by Dutta et al. (2017)].

### 2.3 Cellular model of acute ischemia

To simulate the effects of acute myocardial ischemia at the cell membrane level, more profound changes were made to the O’Hara model [as in Carpio et al. (2022)]. First, a model of the ATP-sensitive potassium current (*I*_*K*(*ATP*)_), which is absent in the O’Hara model, was formulated using the model by Ferrero et al. (1996) adapted to human cardiomyocytes using data from Babenko et al. (1998) by changing the maximum conductance and the sensitivity to intracellular ATP and ADP concentrations ([*ATP*]_*i*_ and [*ADP*]_*i*_, respectively). Secondly, the effects of [*ATP*]_*i*_ and [*ADP*]_*i*_ on ionic pumps were modeled using data from Cortassa et al. (2006) and Terkildsen et al. (2007) by introducing different [*ATP*]_*i*_ and [*ADP*]_*i*_ dependent scaling factors affecting the NaK pump (*I*_*NaK*_), the sarcolemmal calcium pump (*I*_*pCa*_)and the SERCA pump (*I*_*up*_). Thirdly, we introduced the effects of extracellular and intracellular acidosis in the model by applying different multiplicative factors that depend on *pH*_*o*_ and/or *pH*_*i*_ to several pH-dependent currents. Specifically, we used data from Saegusa et al. (2011) to model the effects of acidosis in *I*_*CaL*_; data from Murphy et al. (2011); Watson and Gold (1995) for both *I*_*Na*_ and *I*_*NaL*_; data from Doering et al. (1996); Egger and Niggli (2000) for the sodium/calcium exchanger (*I*_*CaNa*_); and data from Friedrich et al. (1996) to model the effects of acidosis on *I*_*NaK*_. Finally, the effects of LPC) on *I*_*Na*_ and *I*_*NaL*_ were modeled following Gautier et al. (2008).

### 2.4 Simulation of progressive acute ischemia

**FIGURE 1:**
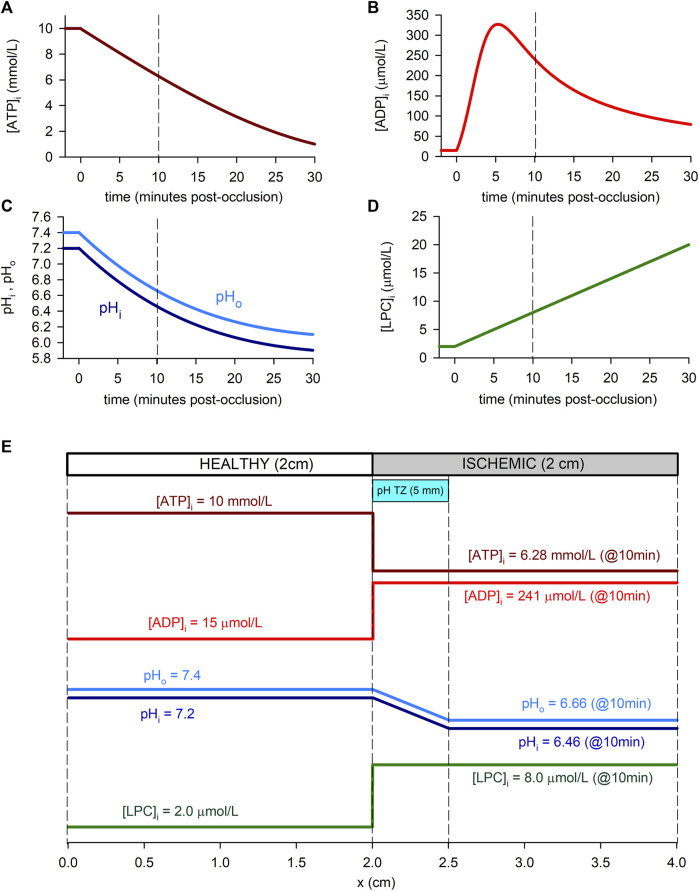
Time course of ischemia-related parameters in the simulated cell (“0D simulations”, panels **(A–D)** and spatial profiles in the simulated tissue (“1D simulations”, panel **(E)**. **(A)**: Intracellular ATP concentration. We adopted the time course reported by Sakamoto et al. (2000) for guinea-pig (at 37°C) with the normoxic value of O’Hara et al. (2011) and Cao et al. (2018). **(B)**: Intracellular ADP concentration, assuming a time course as reported by Terkildsen et al. (2007), based on Befroy et al. (1999) for guinea-pig at 37°C. **(C)**: Intracellular and extracellular pH, adopting the time course for guinea-pig reported by Sakamoto et al. (2000) (at 37°C) and a normoxic value for *pH*_*o*_ by Vaughan-Jones et al. (2009). **(D)**: Intracellular LPC concentration, assumed linear and estimated using data from Gautier et al. (2008) in rat at 22°C and Daleau (1999) for guinea-pig at 30°C. **(E)**: Spatial profiles of the ischemic parameters in relation to the simulated 1D tissue. As an example, values within the ischemic part of the strand correspond to 10 min post-occlusion [dashed lines in panels **(A–D)**]. A pH transition zone (pH TZ) was assumed to be 0.5 cm wide Coronel et al. (1988). pH was assumed to vary linearly within that zone.

Each simulation corresponds to 5 min of normoxia followed by 30 min of acute ischemia. During this 30 min period, hypoxia and acidosis are progressive, and the time evolution of the ischemic parameters is shown in Figures 1A–D. For the [*ATP*]_*i*_ (Figure 1A), the time course reported by Sakamoto et al. (2000) for guinea pig was adopted with a normoxic value of [*ATP*]_*i*_ = 10 mmol/L as in O’Hara et al. (2011) and Cao et al. (2018). Regarding pH (Figure 1C), the time course of *pH*_*i*_ reported by Sakamoto et al. (2000) for guinea pig was assumed. The same time course was adopted for *pH*_*o*_, with a normoxic value of *pH*_*o*_ = 7.4 measured in guinea pig (Vaughan-Jones et al., 2009). For [*LPC*]_*i*_, a linear time course was assumed with a normoxic value of [*LPC*]_*i*_ = 2 μmol/L and a concentration of [*LPC*]_*i*_ = 20 μmol/L at 30 min from the onset of ischemia, estimated from the works by Sobel et al. (1978) and Daleau (1999). As for free intracellular ADP, due to its low basal concentrations, direct measurements of [*ADP*]_*i*_ are not possible and must be estimated using mass-action kinetics principles. In this regard, different profiles of ADP have been derived (Allen and Orchard, 1987; Weiss et al., 1992; Terkildsen et al., 2007). In this work, the ADP profile derived by Terkildsen et al. (2007) has been adopted.

In the 1D simulations, these dynamic changes affected only the “altered tissue”, while in the “normoxic tissue” the values of the parameters remained normal ([*ATP*]_*i*_ = 10 mmol/L [*ADP*]_*i*_ = 15 *μ*mol/L, *pH*_*i*_ = 7.2, *pH*_*o*_ = 7.4, and [*LPC*]_*i*_ = 2 *μ*mol/L). In the case of [*ATP*]_*i*_ [*ADP*]_*i*_ and [*LPC*]_*i*_, these dynamic changes have been imposed in a stepwise manner at the transition between the normal and ischemic tissue, as indicated in the study from Walfridsson et al. (1985) that reports the anoxic border to develop in less than 1 mm. On the contrary, a transition zone of 0.5 cm has been considered for *pH*_*i*_ and *pH*_*o*_ following the model by Ferrero et al. (2003) which was inspired in the studies from Coronel et al. (1988). Figure 1E shows the spatial transition for the cyanotic and the acidosis border used in the simulations.

Due to the large variability of the data present in the literature, additional simulations considering different time-courses and baseline values for the different metabolites were performed for completeness. Results from these simulations are available as part of the Supplementary Figures S1, S3 and will be discussed later.

### 2.5 Stimulation protocol

The cell (in the “0D simulations”) and the first (leftmost) cell of the “normoxic tissue” (in the “1D simulations”) were stimulated during 35 min with a train of pulses 0.5 milliseconds in width and twice normoxic diastolic threshold in amplitude, with a frequency of 1 Hz (unless otherwise stated). The frequency of 1Hz was chosen since most of the experimental studies found in the literature were conducted at this frequency. However, to test the sensitivity of the model to heart rate, additional simulations were carried out in which the cell was paced at different frequencies: i) a quiescent cell (0 beats per minute, bpm), ii) a bradycardic situation (30 bpm), iii) our control heart rate (60 bpm), iv) a mild tachycardia (120 bpm), and v) a severe tachycardia (180 bpm).

### 2.6 Tissue electrophysiology equations

In the 1D simulations, cardiac tissue was modeled as a continuous fiber with a length *L* of 4 cm. The transmembrane potential (*V*_*m*_), and the extracellular potential (*V*_*o*_), were computed using the bidomain model in two steps (Keller et al., 2010). First, *V*_*m*_ was obtained as the solution of the 1D reaction-diffusion equation:

$$C_{m}\frac{\partial V_{m}}{\partial t}=D_{V}\frac{\partial^{2}V_{m}}{\partial x^{2}}-I_{\mathrm{ion}}+I_{\mathrm{stm}}$$ (1)

where *D*_*V*_ is the effective tissue conductivity, *C*_*m*_ is the specific membrane capacitance, *I*_*ion*_ is the transmembrane ionic current density (sum of the transmembrane currents included in the O’Hara model), and *I*_*stm*_ is the stimulation current density. The extracellular potential is governed by the following partial differential equation

$$\frac{\partial^{2}V_{o}}{\partial x^{2}}=-\frac{1}{1+\lambda }\frac{\partial^{2}V_{m}}{\partial x^{2}}$$ (2)

where *λ* is the extracellular to intracellular conductivity ratio. Eqs 1, 2 are subject to non-conduction boundary conditions at both cable ends.

$$\frac{\partial V_{m}}{\partial x}=0,\;at\;x=0\;and\;x=L$$ (3)

$$\frac{\partial V_{o}}{\partial x}=0,\;at\;x=0\;and\;x=L$$ (4)

In the “0D simulations”, Eq 1 without the diffusion term was used:

$$C_{m}\frac{dV_{m}}{dt}=-I_{\mathrm{ion}}+I_{\mathrm{stm}}$$ (5)

### 2.7 Computation of the injury current

One of the interests of this study was to investigate on the characteristics of the “injury current” which flows between the altered and normal tissue during acute ischemia, and to evaluate its potential role in arrhythmogenesis during acute myocardial ischemia (Janse and Kléber, 1981; Coronel et al., 1991). For this purpose the intracellular injury current, $I_{i}^{\mathrm{inj}}$ , was computed through the following differential equation

$$I_{i}^{\mathrm{inj}}=D_{V}\frac{\partial^{2}V_{m}}{\partial x^{2}}.$$ (6)

This equation is computed as a postprocessing step in the simulations.

### 2.8 Potassium transport equations

To model the dynamic changes in extracellular potassium concentration $\left({\left[K^{+}\right]}_{o}\right)$ , in the “1D simulations” we used a modified version of the Nernst-Planck equation (Niederer, 2013) to formulate a transport equation for *K* ^+^. The equation accounts for diffusion, electromigration, and also for the local contribution of transmembrane ionic currents and wash-out:

$$\frac{\partial {\left[K^{+}\right]}_{o}}{\partial t}=D_{K}\frac{\partial }{\partial x}\left(\frac{\partial {\left[K^{+}\right]}_{o}}{\partial x}+\frac{F}{RT}{\left[K^{+}\right]}_{o}\frac{\partial V_{o}}{\partial x}\right)+\frac{A_{c}}{Fv_{o}}\underset{x}{\sum }I_{Kx}+\frac{{\left[K^{+}\right]}_{b}-{\left[K^{+}\right]}_{o}}{\tau_{wo}},$$ (7)

where *D*_*K*_ is the extracellular potassium diffusion coefficient, *F* is the Faraday’s constant, *R* is the gas constant, *T* is the absolute temperature, *V*_*o*_ is the extracellular potential, *A*_*c*_ is the cell surface, *v*_*o*_ is the volume of the extracellular compartment, *∑I*_*Kx*_ the total transmembrane potassium current (*I*_*Kr*_ + *I*_*Ks*_ + *I*_*to*_ + *I*_*K*1_ + *I*_*K*(*ATP*)_ + *I*_*Kb*_ + *I*_*CaK*_ - 2*I*_*NaK*_), ${\left[K^{+}\right]}_{b}$ the bulk extracellular potassium concentration present in the blood stream, and *τ*_*wo*_ is the time constant associated with potassium wash-out (from the extracellular clefts to the blood flow). Eq. 7 is subject to no-diffusion boundary conditions at the cable ends

$$\frac{\partial {\left[K^{+}\right]}_{o}}{\partial x}=0,\;at\;x=0\;and\;x=L.$$ (8)

In the “0D simulations”, Eq. 7 without the diffusion term was used:

$$\frac{d{\left[K^{+}\right]}_{o}}{dt}=\frac{A_{c}}{Fv_{o}}\underset{x}{\sum }I_{Kx}+\frac{{\left[K^{+}\right]}_{b}-{\left[K^{+}\right]}_{o}}{\tau_{wo}},$$ (9)

### 2.9 Computation of potassium flux rates

One of the objectives of this study was to compute the contribution of each individual sarcolemmal current to extracellular potassium accumulation. For this purpose, we define the potassium flux rate (K_*FRx*_) generated by a particular potassium current *I*_*Kx*_ as the rate of increase of ${\left[K^{+}\right]}_{o}$ directly provoked by that current (averaged in a certain time interval, corresponding to four beats unless otherwise stated). A positive value of K_*FRx*_ corresponds to an efflux, and a negative value to an influx.

From Eq. 9 (particularized for a specific potassium current *I*_*Kx*_), the value of K_*FRx*_ may be computed as follows:

$$K_{\mathrm{FRx}}\left(t\right)=\frac{{\left[K^{+}\right]}_{o,\left(t+4\cdot BCL\right)}-{\left[K^{+}\right]}_{o,\left(t\right)}}{4\cdot BCL}=\frac{1}{4\cdot BCL}\frac{A_{c}}{Fv_{o}}\int_{t}^{t+4\cdot BCL}I_{Kx}dt$$ (10)

where BCL (Basic Cycle Length) is the stimulation period (1,000 milliseconds, unless otherwise stated).

The total potassium flux rate (K_*FRT*_) is given by:

$$K_{\mathrm{FRT}}=\underset{x}{\sum }K_{\mathrm{FRx}}$$ (11)

### 2.10 Numerical and computational methods

The “0D simulations” were performed with a custom-made script in MATLAB (MathWorks, Natick, MA) using an adaptive time step method. On the contrary, for the “1D simulations”, Eqs 1–7 were solved with an in-house C code using the operator splitting numerical scheme together with the explicit Euler method. Eq. 2 was integrated with custom-made software routines in MATLAB as a postprocessing step assuming a value of *λ* = 3.647 from Niederer et al. (2011). These simulation codes are available upon request.

For the “1D simulations”, a constant time step of Δ*t* = 0.02 m and a space discretization of Δ*x* = 0.25 mm were used. The parameters used in the simulations are shown in Table 1. The effective tissue conductivity was assumed to be *D*_*V*_ = 0.0026 m that gives a conduction velocity of 70 cm/s in normoxic tissue (Taggart et al., 2000). The extracellular potassium diffusion coefficient *D*_*K*_ = 1.5 ⋅ 10^–8^ cm^2^/s was taken from Niederer (2013). However, additional simulations were conducted at different values of *D*_*V*_ and *D*_*K*_ to test the influence that electrotonic coupling and ${\left[K^{+}\right]}_{o}$ diffusion have on the formation of the ${\left[K^{+}\right]}_{o}$ border zone. To estimate the value of the wash-out time constant in normoxia, we conducted simulations and chose a value for *τ*_*wo*_ that replicated the extracellular potassium transients observed experimentally in normoxia (Kunze, 1977; Hotokebuchi et al., 1987). An infinite value was assigned to *τ*_*wo*_ in the “altered tissue” (in “1D simulations”) and in the ischemic cell (in the “0D simulations”) to mimic the lack of wash-out in ischemia.

**TABLE 1:** Model parameters for the electrophysiology and transport equations.

Parameter	Definition	Value	Unit
*C*_*m*_	Membrane specific capacitance	1.0	*μ*F/cm^2^
*L*	Cable length	4.0	cm
*D*_*V*_	Effective tissue conductivity	0.0026	mS
*F*	Faraday constant	96,486	C/mol
*A*_*c*_	Cell capacitive surface	0.0152	mm^2^
*v*_*o*_	Extracellular cleft volume	13.3	*μ*m^3^
$S_{v}^{i}$	Intracellular surface to volume ratio	363.6	mm^−1^
$S_{v}^{e}$	Extracellular surface to volume ratio	127.3	mm^−1^
*D*_*K*_	${\left[K^{+}\right]}_{e}$ diffusion coefficient	1.5⋅10^–8^	cm^2^/ms
${\left[K^{+}\right]}_{b}$	${\left[K^{+}\right]}_{e}$ bulk concentration	5.4	mM
*τ*_*wo*_	Normoxic wash-out time constant	15	s

The accuracy of the 1D numerical simulations was verified against simulations that employed smaller space and time discretizations of Δ*x* = 0.125 mm and Δ*t* = 0.01 m respectively. Results indicate that reducing the space discretization and time step to these values lead to an increase in the conduction velocity of about 2.0%. However, major results, and main conclusions, of this study obtained with these smaller time and space discretizations are still valid as discussed in the following section and reported in the Supplementary Figure S5.

## 3 Results

### 3.1 Single cell simulations

This section describes the main results related to the “0D simulations”, corresponding to an isolated ventricular cardiac myocyte.

#### 3.1.1 Extracellular potassium accumulation

**FIGURE 2:**
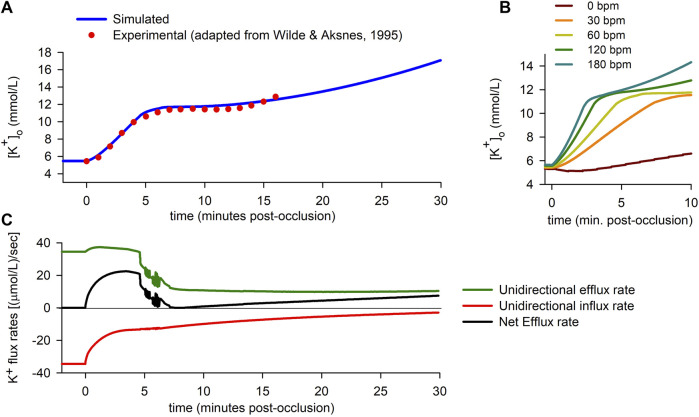
Extracellular potassium accumulation and transmembrane potassium fluxes. **(A)**: Extracellular potassium concentration $\left({\left[K^{+}\right]}_{o}\right)$ vs. time in an isolated ventricular cardiomyocyte subject to progressive ischemia. Solid blue line: simulated result; red circles: experimental data adapted from Wilde and Aksnes (Wilde and Aksnes, 1995). **(B)**: Simulated time course of ${\left[K^{+}\right]}_{o}$ for different heart rates (bpm: beats per minute). **(C)**:Unidirectional potassium efflux rate (green), influx rate (red) and net efflux rate (black) vs. time.

Figure 2 summarizes some of the main findings corresponding to the “0D simulations”. The solid line in Figure 2A shows the time course of ${\left[K^{+}\right]}_{o}$ during 30 min of simulated progressive ischemia in the virtual cardiomyocyte stimulated at 1 Hz. Data were sampled at 1 s intervals to eliminate the small “micro” fluctuations (in the *μmol*/*L* range) in ${\left[K^{+}\right]}_{o}$ during the systole-diastole cycle, and highlight the“macro” behaviour of ${\left[K^{+}\right]}_{o}$ . It can be seen that ${\left[K^{+}\right]}_{o}$ is stable before the onset of ischemia (with a value of 5.4 mmol/L), rises during the first ≈5 min, smoothly reaches a plateau (≈12 mmol/L) that lasts until the 15th minute approximately, and then slowly increases again.

To test the validity of the simulation, results were compared to experimental data published elsewhere (Wilde and Aksnes, 1995). The dots in Figure 2A correspond to adapted experimental data of extracellular potassium accumulation obtained in rabbit hearts subject to acute global ischemia. The data shown in the figure have been shifted +0.9 mmol/L to account for the difference between the normoxic value of ${\left[K^{+}\right]}_{o}$ in Wilde and Aksnes. (1995) (4.5 mmol/L) and in our simulations (5.4 mmol/L). After the 16th minute post-occlusion, the experimentally measured ${\left[K^{+}\right]}_{o}$ increased faster than in our simulation (not shown). In Supplementary Figure S1, a direct comparison of our simulation with multiple experimental recordings reported in different works, corresponding to a variety of animal species and/or heart rates, is shown without any shift in the ${\left[K^{+}\right]}_{o}$ values.

In order to test the soundness and sensitivity of the results to the specific time courses of intracellular ATP and intra/extra-cellular pH, we carried out alternative simulations using different time courses and/or initial normoxic values and/or final ischemic values for said ischemic parameters. The results, which can be found in Supplementary Figure S2, show that, despite of small quantitative differences, the qualitative features of the extracellular *K* ^+^ behavior remain unaltered. As for the effects of different possible time courses of intracellular ADP, its effects are presented and discussed below.

Finally, the effect of heart rate is shown in Figure 2B. The results show that the rate of rise of extracellular potassium increases with heart rate. In the particular case of a quiescent cell, the rise in ${\left[K^{+}\right]}_{o}$ is slow and delayed, following an initial period in which a slight decrease is found.

#### 3.1.2 Potassium flux rates

The increase in ${\left[K^{+}\right]}_{o}$ observed in the simulation and the experiments must be due to an imbalance between potassium efflux and influx that causes a net efflux during the ${\left[K^{+}\right]}_{o}$ rising phase. To further analyze the behaviour of potassium fluxes, we computed the potassium flux rates using Eqs 10, 11 (see “Materials and Methods”). Figure 2C shows the time course of the different total potassium flux rates (separated into “Efflux” for the unidirectional positive fluxes through potassium channels, “Influx” for the unidirectional negative flux through the NaK pump, and “Net Efflux” for the difference).

During the normoxic period, efflux and influx are equal in magnitude [34.5 (*μ*mol/L)/s], so there is no net potassium efflux. As soon as ischemia begins, an imbalance between the potassium efflux and influx quickly arises, with a rapid decrease in the magnitude of the influx rate being coupled to a lesser increase in the efflux rate. The resulting net efflux rise (Figure 2C) during the first ≈4 min post-occlusion provokes the first rising phase in ${\left[K^{+}\right]}_{o}$ Figure 2A.

Just before the fifth minute of ischemia, potassium influx has almost stabilized [at ≈ 13 (*μ*mol/L)/s]. However, the efflux rate suffers an abrupt drop, just prior to showing notorious oscillations [which are due to alternans in the action potential duration (APD), as explained below]. In the following 2 minutes, the efflux keeps decreasing until it almost equals the magnitude of the influx. As a result, the net potassium efflux falls to near zero values, which in turn gives rise to the plateau in ${\left[K^{+}\right]}_{o}$ observed in Figure 2A. After several minutes, the efflux rate stabilizes in a lower value [at ≈ 10.5 (*μ*mol/L)/s], while the magnitude of the influx suffers a slow but sustained decrease, provoking a continuous slow increase in the net potassium efflux (Figure 2C). This gives rise to the secondary increase in ${\left[K^{+}\right]}_{o}$ observed from roughly the 10th minute onwards in Figure 2A.

**FIGURE 3:**
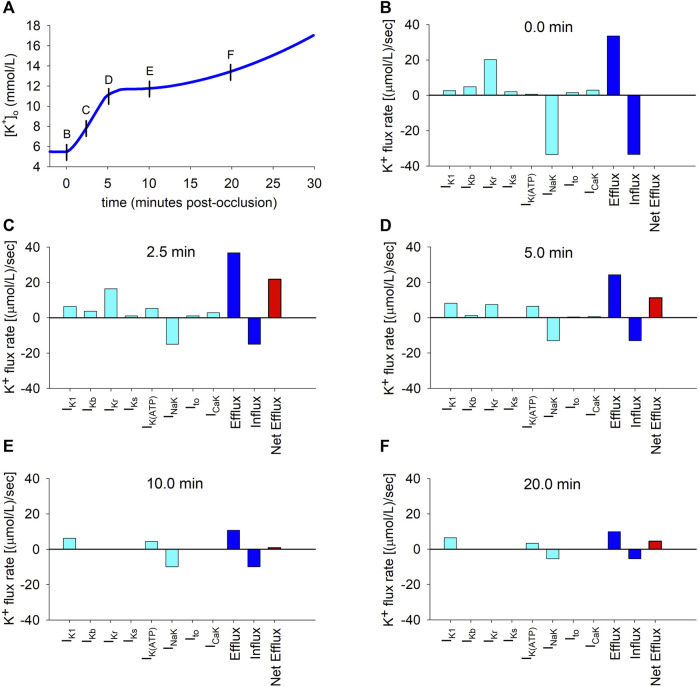
Potassium transmembrane fluxes carried by individual ionic currents. **(A)**: Simulated ischemic ${\left[K^{+}\right]}_{o}$ vs. time in an isolated ventricular cardiomyocyte. **(B–F)**: Individual contributions of potassium currents to potassium transmembrane flux rates. Cyan coloured bars indicate efflux rates (when positive) or influx rates (when negative). Blue bars show total efflux and influx rates. Red bar indicates net efflux rate.

To better understand the causes of the dynamic changes in potassium efflux and influx, the individual contributions of the different potassium transmembrane currents were computed using Eq. (10) at selected time instants depicted by the short vertical lines in Figure 3A. The results are depicted in panels B to F in Figure 3. The coloured bars represent efflux rates (when positive) or influx rates (when negative). The continuous data of the time course of the flux rates corresponding to the individual contributions of all the potassium currents is shown in Supplementary Figure S3.

As shown in Figure 3B, which corresponds to the normoxic situation just before the onset of ischemia, the current carrying the highest potassium efflux is *I*_*Kr*_, with other currents playing a minor role. Unsurprisingly, the NaK pump is the only current that generates a noticeable potassium influx. At this time point, influx and efflux are balanced.

Two and a half minutes later (Figure 3C), the most notorious change is the decrease in the magnitude of the influx rate [from 34.5 to 15.0 (*μ*mol/L)/s] caused by the reduction in the current transported by the NaK pump. Concomitantly, the efflux rates through *I*_*K*1_ and *I*_*K*(*ATP*)_ increase considerably, counteracting the decrease in the *I*_*Kr*_ efflux rate, with changes in other potassium currents being negligible. Thus, the unidirectional potassium efflux increases (from 34.5 to 37.2 (*μ*mol/L)/s). The overall result is an increase in the net efflux [from 0 to 22.2 (*μ*mol/L)/s], which provokes the primary rise in ${\left[K^{+}\right]}_{o}$ shown in Figure 3A.

Five minutes post-occlusion (Figure 3D), the efflux rates through various ion channels have considerably changed. Most noticeably, the efflux generated by *I*_*Kr*_ has strongly diminished and the only currents generating an appreciable potassium efflux are now *I*_*Kr*_, *I*_*K*1_ and *I*_*K*(*ATP*)_. Even if the influx through the NaK pump has very slightly decreased, this important reduction in the efflux provokes a strong decrease in the net efflux rate [now 11.2 (*μ*mol/L)/s]. This is responsible for the decrease in the rate of rise in ${\left[K^{+}\right]}_{o}$ observed at that time in Figure 3A, which in turn begins to generate the plateau in potassium accumulation.

Ten minutes post-occlusion (Figure 3E), when ${\left[K^{+}\right]}_{o}$ has already reached the plateau, only *I*_*K*1_ and *I*_*K*(*ATP*)_ generate potassium efflux, with the efflux of other potassium currents having dropped to zero. This causes a strong decrease in the total efflux compared to Panel D. Meanwhile, the NaK pump has continued decreasing slowly. At this time point, efflux and influx are almost equal [yielding a net efflux rate of 0.9 (*μ*mol/L)/s], which is consistent with the plateau phase in extracellular potassium seen in Figure 3A.

This situation is similar after 20 min of ischemia (Figure 3F), except that the NaK pump influx is further reduced, again generating an imbalance between influx and efflux that gives rise to a net efflux of 4.5 (*μ*mol/L)/s and a secondary slow rise in ${\left[K^{+}\right]}_{o}$ shown in Figure 3A.

#### 3.1.3 Ionic currents

**FIGURE 4:**
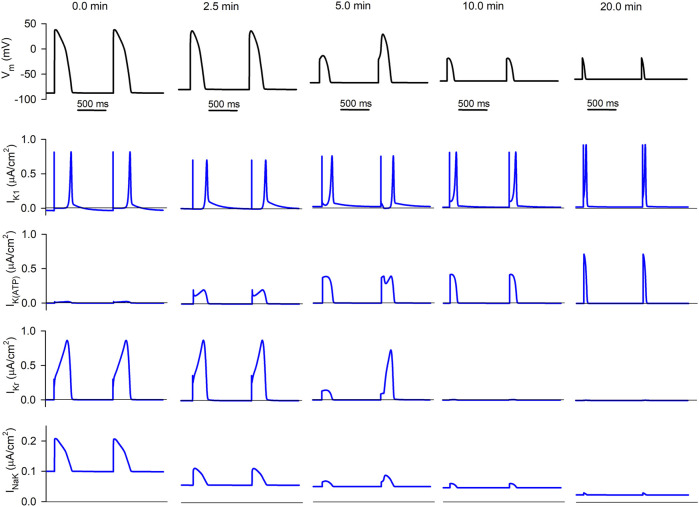
Simulated APs and selected ionic currents in an acutely ischemic isolated ventricular cardiomyocyte. Top row (black traces): consecutive APs 0.0, 2.5, 5.0, 10.0, and 20.0 min after the onset of progressive ischemia. Lower rows (blue traces): inward rectifier potassium current (*I*_*K*1_), ATP-sensitive potassium current (*I*_*K*(*ATP*)_), rapid delayed rectifier potassium current (*I*_*Kr*_), and sodium/potassium pump (*I*_*NaK*_), respectively.

To further investigate the causes of these changes in efflux and influx as ischemia develops, we plotted the time course of APs and selected potassium currents at the time instants discussed above. The top row in Figure 4 shows how APs change as ischemia progresses. Resting membrane potential becomes increasingly less negative (a direct consequence of the increase in ${\left[K^{+}\right]}_{o}$ ), APD decreases (due to hyperkalemia and also to the partial activation of the ATP-sensitive potassium channels), and the cell has become almost non-excitable by the 10th minute. Moreover, AP alternans exist in the fifth minute post-occlusion.

The second row shows the progressive evolution of *I*_*K*1_. Basically, its diastolic value keeps increasing as ischemia develops due to the hump in its current-voltage relationship that implies a higher current at membrane potentials moderately higher than the potassium Nernst potential (*E*_*K*_). This causes the increase in its associated efflux seen in Figures 3D–F.

The third row represents the evolution of *I*_*K*(*ATP*)_. While it is almost non-existent in normoxia, its amplitude keeps increasing in ischemia (due to the decrease in the intracellular ATP/ADP ratio). Although its duration decreases with time (due to the reduction in APD), the area below the curve (proportional to the efflux generated by the current) is almost constant.

The fourth row shows the progression of *I*_*Kr*_ (which generates the highest potassium efflux in normoxia) with ischemia. Its duration has decreased after 2.5 min of ischemia, thus reducing the efflux, and it has almost vanished 10 min after the onset of ischemia (due to the cell being almost non-excitable at this point). Five minutes post-occlusion, AP alternans cause an alternation in the amplitude and duration of *I*_*Kr*_, which is very low in the short AP compared to the long one. Further effects of AP alternans in the time course of ${\left[K^{+}\right]}_{o}$ will be discussed below.

Finally, the lower row corresponds to the NaK pump current. The decrease in intracellular ATP levels continuously reduces the magnitude of the current, thus reducing the influx of potassium as was apparent in Figures 3B–F.

#### 3.1.4 Action potential alternans and their effect in potassium accumulation

Once we identified the causes of the triphasic nature of extracellular potassium accumulation in regards to potassium fluxes, we further analyzed the influence of the dynamically changing AP morphology during ischemia on the time course of ${\left[K^{+}\right]}_{o}$ .

**FIGURE 5:**
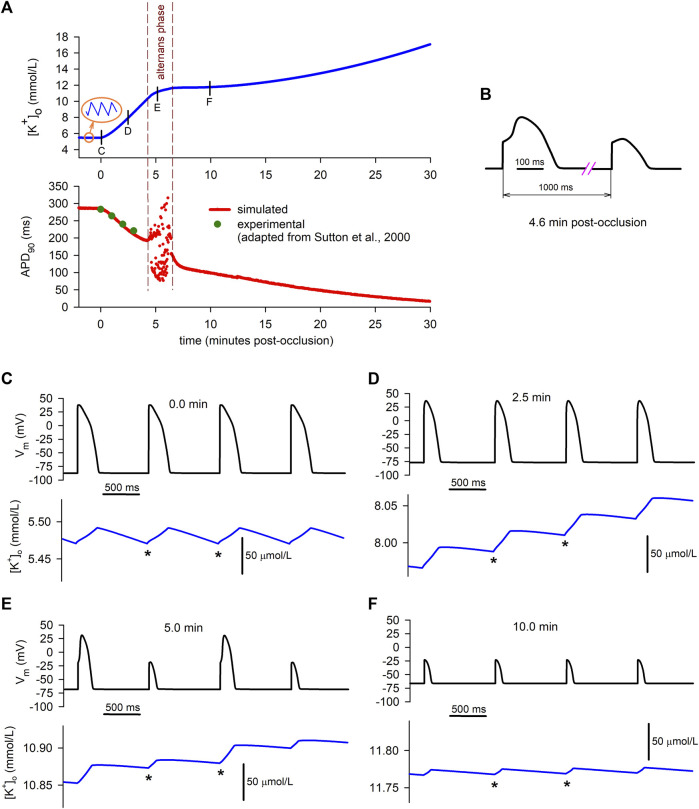
Relationship between simulated extracellular potassium concentration and APs. **(A)**: time course of ${\left[K^{+}\right]}_{o}$ (upper graph) and APD at 90% repolarization (lower graph), showing the AP alternating phase (dashed vertical lines). **(B)**: simulated AP alternans corresponding to the 4.6 min mark. The time axis has been broken to enhance the shape of the “short” and “long” APs. **(C–F)**: Action potentials (upper black traces) and ${\left[K^{+}\right]}_{o}$ (lower blue traces) showing the “micro” fluctuations in extracellular potassium concentration. The * symbols indicate diastolic minimum potassium levels.

The data depicted in red in the lower graph in Figure 5A represents the time course of the APD at 90% repolarization (*APD*_90_) during the 30 min period of simulated ischemia at the same time scale as ${\left[K^{+}\right]}_{o}$ (upper blue graph). Immediately after the onset of ischemia, *APD*_90_ begins to decline, as expected. To test the validity of these results, the simulated values were compared to experimental results published elsewhere. The four green dots in the lower graph of Figure 5A show Monophasic Action Potential (MAP) duration values (adapted by adding 32 m to account for the basal difference with our *APD*_90_ values) measured in acutely ischemic human hearts *in-vivo* (Sutton et al., 2000).

As shown in the lower graph of Panel A, between the fourth and the sixth minute of ischemia (approximately), *APD*_90_ values begin to oscillate, indicating that the cell is developing electrical alternans. This alternans phase is delimited by the dashed vertical lines. Figure 5B shows the detail of the waveform of two consecutive alternating APs obtained in our simulations (at the 4.6 min mark). At the beginning of this phase, alternans with a 2:2 pattern (i.e., long–short–ong–short) are predominant, but shortly afterwards more complex patterns arise (e.g., long–short–short–long–long–short–long).

As the dashed vertical lines indicate, the period of occurrence of alternans matches the smooth transition from the primary rising phase of ${\left[K^{+}\right]}_{o}$ to the plateau, suggesting that the alternans themselves may be responsible for the gradual deceleration of the extracellular potassium rise and the genesis of the plateau. To confirm this hypothesis, APs at selected time intervals spanning four consecutive APs were plotted together with ${\left[K^{+}\right]}_{o}$ . At these short time scales, ${\left[K^{+}\right]}_{o}$ clearly shows the “micro” fluctuations caused by the systole-diastole cycles (inset in Panel A top graph), as clearly seen in the lower (blue) plots of Panels C through F.

During normoxia (Figure 5C), APs do not alternate at all, and potassium currents are balanced in a way that the diastolic minimum potassium levels (DmKL, indicated by the * signs) reach the same value at the end of each diastolic period. This is consistent with the normoxic stable behaviour of the “macro” ${\left[K^{+}\right]}_{o}$ with zero net efflux shown in Figures 2C, Figure 3B.

On the contrary, at the 2.5 min mark in the ischemic cell (Figure 5D), the positive net potassium efflux seen in Figures 2C, Figure 3C is related to a continuous increase in the DmKL from one AP to the following one. Consequently, the “macro” ${\left[K^{+}\right]}_{o}$ rapidly increases. However, this trend is attenuated when alternans begin to appear. Five minutes post-occlusion (Figure 5E), when the “macro” ${\left[K^{+}\right]}_{o}$ is decelerating (Figure 5A), the difference between consecutive DmKL values diminishes in relation to Panel D. The reason is that the “short” APs originate lower positive shifts in the “micro” ${\left[K^{+}\right]}_{o}$ fluctuations due to the fact that *I*_*Kr*_, the strongest efflux generator at the 2.5 min mark, almost vanishes in the “short” APs (see Figure 4). This is because a) it is a systolic current that is only active during the AP, which is now short, and b) because the driving force for potassium ions is smaller due to the lesser peak of the short AP. Therefore, the average potassium net efflux rate diminishes (Figures 2C, 3D), causing a reduction in the slope of the “macro” ${\left[K^{+}\right]}_{o}$ that eventually causes the plateau to appear (upper Panel A).

Finally, 10 min after the onset of ischemia (Figure 5F), only short APs are present in the cell due to its partial loss of excitability provoked by an already elevated value of ${\left[K^{+}\right]}_{o}$ (see Panel A). In these circumstances, the low positive shifts in the “micro” ${\left[K^{+}\right]}_{o}$ fluctuations are almost equal for consecutive beats, thus the “macro” ${\left[K^{+}\right]}_{o}$ remains almost constant (as seen in the upper graph of Panel A).

### 3.2 Tissue simulations

This section describes the main results related to the “1D simulations”, corresponding to a virtual 4 cm strand subject to normal conditions in its first 2 cm (the “normoxic tissue”) and to progressive acutely ischemic conditions in the last 2 cm (the “altered tissue”).

#### 3.2.1 Extracellular potassium accumulation

**FIGURE 6:**
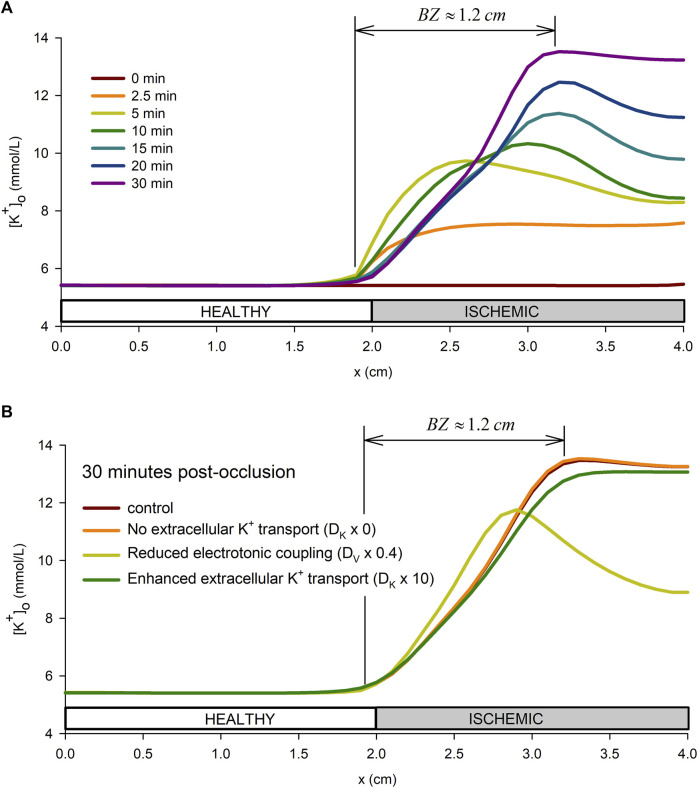
Spatial profile of extracellular potassium accumulation. **(A)**: spatial profile in the tissue preparation at different times from the onset of ischemia. **(B)**: influence of the tissue conductivity and extracellular potassium diffusion coefficient on the extracellular potassium spatial profile at 30 min from the onset of ischemia.

Figure 6A shows the spatial profiles of ${\left[K^{+}\right]}_{o}$ in the tissue preparation from just before and up to 30 min after the onset of ischemia. This profile increases monotonically in the altered tissue for the first 2 minutes post-occlusion, to then adopt a non-monotonic profile with the spatial peak increasing with time and moving toward the center of the altered tissue. These changes in ${\left[K^{+}\right]}_{o}$ also affect a small region in the distal part of the “normoxic” tissue. The position of the peak value of ${\left[K^{+}\right]}_{o}$ stabilizes after 15 min of the onset of ischemia, defining an ischemic BZ of approximately 1.2 cm, in agreement with experimental results (Hill and Gettes, 1980; Coronel et al., 1991). The slope of the rising phase of the ${\left[K^{+}\right]}_{o}$ keeps increasing until minute five post-occlusion before starting to decrease, while the spatial peak of the ${\left[K^{+}\right]}_{o}$ profile moves toward the center of the ischemic zone. Around minute 15, the rising phase of the ${\left[K^{+}\right]}_{o}$ profile has become almost linear, and the location of the peak has stabilized. From this time onwards, the peak keeps rising together with the concentration in the CIZ until almost reaching a plateau by minute 30. A video with the evolution of the ${\left[K^{+}\right]}_{o}$ profile with time is available in the Supplementary Movie SM1.

To test the sensitivity of the results to the time-course of [*ADP*]_*i*_ and the spatial extension of the pH transition zone, simulations considering the ADP time-course proposed by Weiss et al. (1992) were performed for three different pH transition zone sizes (0 cm, 0.5 cm, and 1 cm). Results from the simulations are shown in Supplementary Figure S4. The results show that the ADP data from Weiss et al. (1992) leads to a lower rate of rise of ${\left[K^{+}\right]}_{o}$ during the primary rising phase, which delays the potassium plateau with respect to our control simulations based on the ADP data from Terkildsen et al. (2007). However, the results are qualitatively similar. Indeed, a plateau is reached when AP alternans flatten the curve, the same as in our main simulations (compare left and right curves in Supplementary Figure S4B, C). As for the ${\left[K^{+}\right]}_{o}$ spatial profiles, they are affected by the time-course of ADP (the size of the ${\left[K^{+}\right]}_{o}$ border zone in particular), with changes in the order of a 10%, with the pH transition zone affecting less. However, the time evolution described in the previous paragraphs is observed to be the same for both of the considered ADP time-courses. Furthermore, simulations performed with a mesh size of 0.125 mm showed no difference in the results with respect to those obtained with a mesh size of 0.25 mm (see Supplementary Figure S5).

Furthermore, to better understand the mechanisms behind the formation of the BZ, a sensitivity analysis of the effect of the tissue conductivity and the potassium diffusion coefficient was performed. Together with the control simulation, the following combination of parameters were considered: i) The tissue conductivity, *D*_*V*_, was reduced by 20%, by 60%, and by 80% down to 0.0005 m (conduction velocity of 30 cm/s) while keeping the extracellular potassium diffusion coefficient, *D*_*K*_, at the control value in Table 1; ii) the extracellular potassium diffusion coefficient, *D*_*K*_, was multiplied by 0 (no extracellular potassium transport i.e., *D*_*K*_ = 0.0), by 10, and by 100 while keeping the effective tissue conductivity to the value in Table 1. Results of these simulations are show in Supplementary Figure S6, whereas Figure 6B shows only results for four representative cases. Results show that increasing *D*_*K*_ reduces the value of ${\left[K^{+}\right]}_{o}$ in the central ischemic zone and enlarges the BZ by increasing the region in the distal part of the normoxic tissue affected by ischemia. This leads to a smoother transition between the normoxic and the CIZ. Conversely, reducing *D*_*V*_ makes the BZ smaller, reduces the maximum ${\left[K^{+}\right]}_{o}$ , and leads to a biphasic ${\left[K^{+}\right]}_{o}$ spatial profile. The reduction in the maximum ${\left[K^{+}\right]}_{o}$ and the biphasic behavior in the spatial profile are caused by an early appearance of AP alternans in the border zone and conduction block in the CIZ as *D*_*V*_ decreases. The early appearance of AP alternans cause a premature ending of the rising phase, whereas the absence of systolic current in the CIZ after conduction block causese a reduction in the extracellular *K* ^+^ efflux responsible for the biphasic behavior in the spatial profile. Most importantly, results indicate a larger influence of the electrotonic coupling compared to extracellular potassium transport in the formation of the BZ. Indeed, the figure shows that a 60% reduction in tissue conductivity implies a 40% reduction in the size of the BZ (from 1.2 cm to 0.72 cm), whereas a 10 fold increases in *D*_*K*_ only increased the size of the BZ in a 10% (from 1.2 cm to 1.35 cm). In addition, the total suppression of extracellular potassium transport left the BZ almost unaltered (see Figure 6B).

**FIGURE 7:**
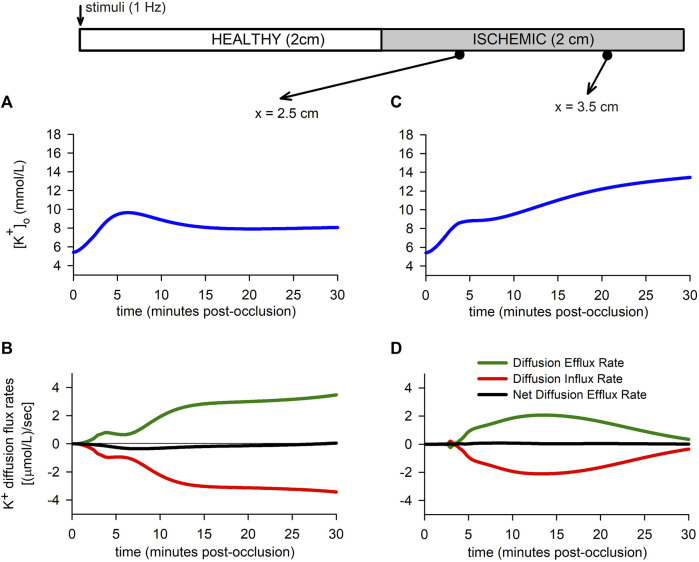
Extracellular potassium flux rates. **(A,B)**: Extracellular potassium accumulation [Panel **(A)**] and extracellular potassium diffusion flux rate [Panel **(B)**] for a point located in the BZ. **(C,D)**: Extracellular potassium accumulation [Panel **(C)**] and extracellular potassium diffusion flux rate [Panel **(D)**] for a point located in the CIZ.

To better understand the role of extracellular potassium transport in the ${\left[K^{+}\right]}_{o}$ accumulation, the efflux, influx and net efflux diffusion rates as a function of time are depicted in Figure 7 for two positions in the tissue, namely *x* = 2.5 cm in the BZ, and *x* = 3.5 cm in the CIZ. The figure shows that the net diffusion efflux rate is four orders of magnitude smaller than the net efflux associated with the various ion channels shown in Figure 3. The figure also shows that net diffusion efflux rate results non-zero when the ${\left[K^{+}\right]}_{o}$ experiences a maximum (see Figures 7A, B), or during the transition from the first rising phase to the second rising phase (see Figures 7C, D). However, even in this case, the net diffusion efflux is much smaller than the transmembrane efflux. From the analysis of Figure 7 it emerges that the evolution in the spatial ${\left[K^{+}\right]}_{o}$ profile is associated with a heterogeneous local potassium accumulation in the altered region.

#### 3.2.2 Electrophysiological manifestations of the extracellular potassium accumulation in tissue

**FIGURE 8:**
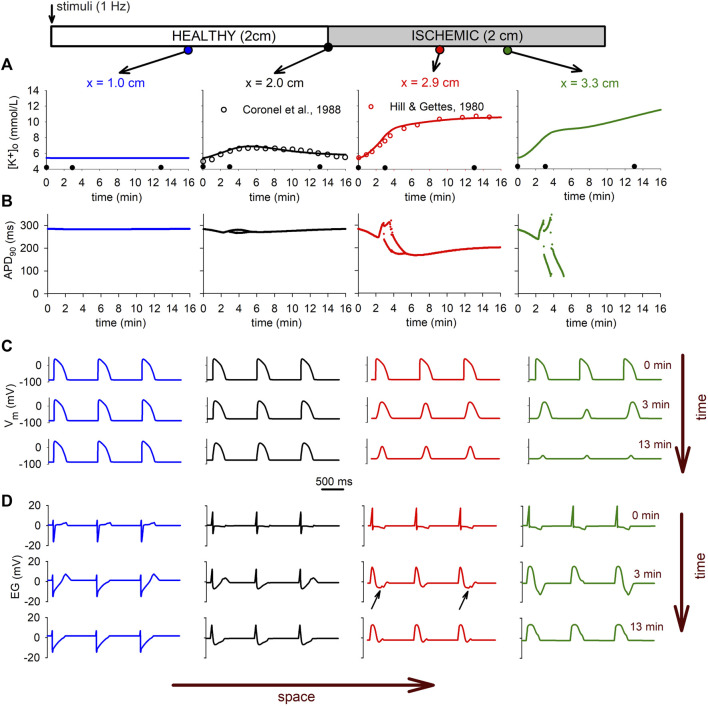
Electrophysiological manifestations of the extracellular potassium accumulation in tissue. **(A)**: time course of the ${\left[K^{+}\right]}_{o}$ at different locations in the tissue. Model results at the BZ are compared with experimental results (Coronel et al., 1988). **(B)**: Evolution of the *APD*_90_ during acute ischemia in different locations. **(C)** Evolution of the AP during ischemia at different positions in the tissue. **(D)**: Changes in the electrograms with ischemia at different locations in the tissue. Arrows indicate the depression in the ST segment due to ischemia in the altered region.

Figure 8 shows the ${\left[K^{+}\right]}_{o}$ time profile at different locations within the tissue, together with the evolution of the APs and the electrograms during the first 16 min of ischemia. The time profile of ${\left[K^{+}\right]}_{o}$ (Panel A) changes according to the position within the tissue. While in the unaltered region the ${\left[K^{+}\right]}_{o}$ remains constant, as expected, in the BZ (position *x* = 2 cm) the time profile shows a non-monotonic behavior, in good agreement with the experimental results reported by Coronel et al. (1988) in regionally ischemic pig hearts. As moving from the BZ towards the center of the ischemic zone (positions *x* = 2.9 cm and *x* = 3.3 cm), the ${\left[K^{+}\right]}_{o}$ time profile evolves toward the characteristic triphasic shape shown in Figure 2A for the isolated cell, and in good agreement with experimental data reported by Hill and Gettes (1980). While the local triphasic behavior of ${\left[K^{+}\right]}_{o}$ is associated with the ionic mechanisms described in the previous sections, the heterogeneity in the ${\left[K^{+}\right]}_{o}$ time profile within the tissue appears to be mainly related to the electrotonic coupling as demonstrated in Figure 6B.

The heterogeneity observed in the ${\left[K^{+}\right]}_{o}$ time profile is also observed in the APD (Figure 8B) and in the APs themselves (Figure 8C). Within the CIZ (*x* = 3.3 cm) the AP shows changes in morphology associated with the alterations due to ischemia, with alternans developing after the 2.5 min mark, followed by total loss of excitability after the 5.5 min mark. Concomitant changes are observed in the proximal side of the CIZ (*x* = 2.9 cm). Here, a certain degree of alternation is also observed, though the cells remain electrically responsive during the whole simulation period. At the proximal side of the BZ (*x* = 2.0 cm), AP alternans are hardly noticed and the only changes in the AP morphology happen due to alterations associated with ischemia.

These electrophysiologic changes are clearly reflected in the electrograms depicted in Figure 8D for different positions in the tissue. The electrograms show the typical depression in the ST segment due to ischemia in the altered region (small black arrows in Figure 8D), whereas the alternans in the AP are reflected in alternans in the T-wave (see Figure 8D right panel). The conduction block in the CIZ at minute 13 is evident by the total suppression in of the T-wave in all electrograms, and the monophasic electrogram at the CIZ (*x* = 3.3 cm).

#### 3.2.3 Injury current during acute regional ischemia

**FIGURE 9:**
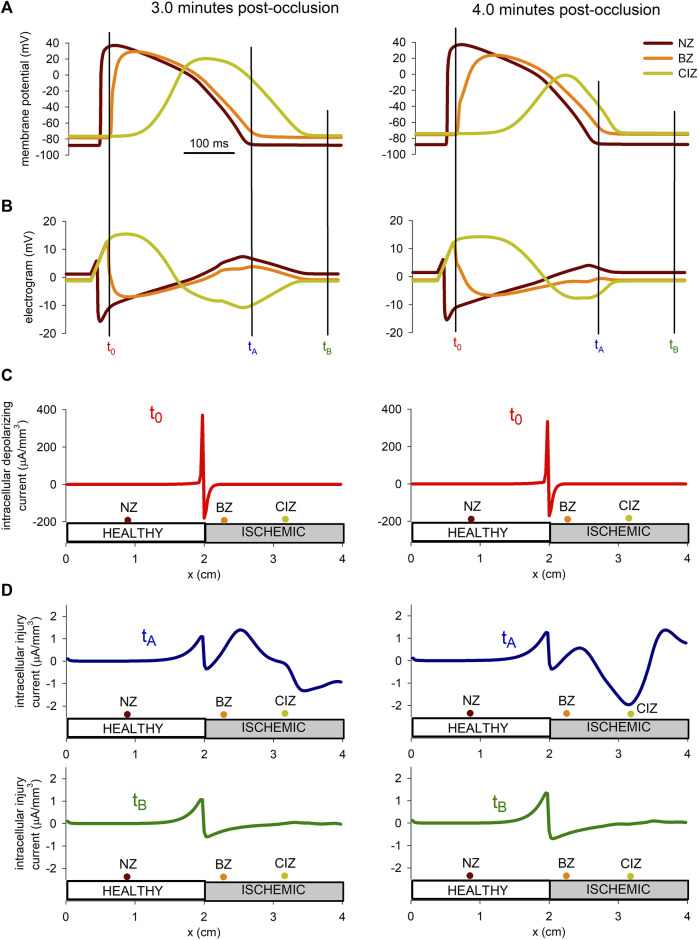
Injury current during acute regional ischemia. **(A)**: Action potentials at three different locations within the cable at two instances after the onset of ischemia. **(B)**: Simulated electrograms at the same positions and time instants as in panel **(B)**. **(C)** Intracellular depolarizing current at the moment the depolarizing front enters the ischemic region. **(D)** Intracellular injury current at the moment of deepest negative T-wave in the ischemic zone and during the diastolic phase.

The hypothesis that during acute ischemia, a current flowing between the ischemic and the normal tissue, a current of injury, may cause depolarization of the normal cells near the border zone has been proposed in several studies (Janse et al., 1980; Coronel et al., 1991). To explore this hypothesis more in detail by means of numerical simulations, the injury current in the tissue at 3 min and 4 min after the onset of ischemia were computed. The upper panel in Figure 9 show the AP at three position in the tissue located at the normal zone (*x* = 0.9 mm), the border zone (*x* = 2.3 mm), and the central ischemic zone (*x* = 3.2 mm), whereas panel B shows the electrograms at the same positions. The instant *t*_*A*_ has been chosen in correspondence with the end of repolarization of the cell in the normal zone, coincident with the point of deep negative T wave in the electrogram recorded in the ischemic zone. On the contrary, *t*_*B*_ corresponds to an instant of complete repolarization in the three positions. Panel C shows the depolarization current density for the stimulations at 3 and 4 min after the onset of ischemia, at the instant when the propagation front is entering the altered region (*x* = 2 cm). Panel D depicts the intracellular injury currents at instants *t*_*A*_ and *t*_*B*_ at 3 min and 4 min after the onset of ischemia.

The results in Figure 9 show that the magnitude of the depolarization current, required to generate an ectopic beat, is about eight times larger than the magnitude of the injury current flowing from the ischemic to the normal zone.

## 4 Discussion

### 4.1 Main findings

The main findings of our work can be summarized as follows.

1. Ischemia-induced extracellular potassium accumulation and its characteristic triphasic time course are an intrinsic feature of the isolated ischemic cell (Figure 2). Though modulated by diffusion of potassium within the tissue, they are not directly caused by it.
2. The primary (initial) rising phase of ${\left[K^{+}\right]}_{o}$ is mainly caused by a strong progressive reduction of potassium influx (due to the partial inhibition of the NaK pump) concomitant with a lesser increase in the efflux (due to enhancements in *I*_*K*1_ and *I*_*K*(*ATP*)_).
3. The smooth transition to the plateau phase of ${\left[K^{+}\right]}_{o}$ is mainly caused by the appearance of electrical AP alternans, which are in turn provoked by the combination of a lower cell excitability together with an enhanced *I*_*K*(*ATP*)_.
4. Once the alternating period is over, the plateau phase of ${\left[K^{+}\right]}_{o}$ is mainly caused by the predominantly short APs that stabilize potassium efflux.
5. The secondary rise in ${\left[K^{+}\right]}_{o}$ is mainly caused by a stabilization of potassium efflux (due to enhanced *I*_*K*1_ and *I*_*K*(*ATP*)_) concomitant with a slower decrease in the magnitude of the influx carried by a depressed NaK pump.
6. The results obtained in a regionally ischemic tissue explain the progressive appearance of a potassium BZ of approximately 1.2 cm within the proximal end of the ischemic zone.
7. The simulations suggest that the ${\left[K^{+}\right]}_{o}$ spatial profile is mainly due to the electrotonic coupling that modulates the ionic currents and the subsequent net potassium efflux, with the extracellular potassium transport playing a minor role.
8. Results from the simulations suggest that the injury current associated with the deep negative T-wave in the ischemic zone may not be sufficient to depolarize healthy cells in the normal zone. In addition, part of the outward injury current occurs inside the altered tissue, where the cells are still within the effective refractory period.

While findings 1, 2, 4, 6, and 8 confirm previous hypothesis and results from previous modeling works regarding the extracellular *K* ^+^ accumulation during acute ischemia, findings 3, 4, 5, and 7 identify novel mechanisms behind the time course of *K* ^+^ accumulation and its spatial heterogeneity during acute ischemia. These findings are discussed in detail in the following sections.

### 4.2 Comparison with experimental data

The results obtained with our model show a very high degree of agreement with experimental data. This applies both to the time course of ${\left[K^{+}\right]}_{o}$ and to the APD. As seen in Figure 2A, the time course of ${\left[K^{+}\right]}_{o}$ during the primary rise, the plateau and the initial phase of the secondary rise is quantitatively consistent with experimental data from rabbit hearts subject to global acute ischemia (Wilde and Aksnes, 1995). As shown in Supplementary Figure S1, other experiments conducted in animal hearts also show qualitative similarities with ours, both in globally ischemic hearts (Weiss and Shine, 1981; Weiss and Shine, 1982a; Weiss and Shine, 1982b; Kléber et al., 1987; Wilde et al., 1990) and in regionally ischemic hearts (Hill and Gettes, 1980; Hirche et al., 1980; Friedrich et al., 1981; Watanabe and Gettes, 2016; Watanabe and Gettes, 2018). Although the slope of rise in both phases and the time to reach the plateau vary with species and experimental conditions, the qualitative resemblance between experiments and our simulations stands out.

Experimental evidence shows that the rate of rise of ${\left[K^{+}\right]}_{o}$ depends on the frequency with which the heart is stimulated (Weiss and Shine, 1982a; Weiss and Shine, 1986; Weiss et al., 1989; Wilde and Aksnes, 1995; Kanda et al., 1997). To test if our model correctly reproduced this dependency, we stimulated our virtual myocyte at different frequencies and monitored the time course of ${\left[K^{+}\right]}_{o}$ . The results, shown in Figure 2B, are in qualitative agreement with the experimental results (Weiss and Shine, 1982a; Wilde and Aksnes, 1995). As observed in experimental recordings, the model predicts a slower potassium rise at lower heart rates. In particular, if the cell is not stimulated at all (0 beats per minute), the level of ${\left[K^{+}\right]}_{o}$ transiently and slightly decreases below the normoxic value before eventually increasing, which is also found in the experiments (see Figure 2 in (Wilde and Aksnes, 1995) and Figure 5 in (Weiss and Shine, 1982a)).

As for the evolution of the APD, data from the literature obtained in ischemic human hearts is scarce because it is very difficult to obtain. The data from Sutton et al. (2000), plotted in Figure 5A (lower panel), was recorded during 3 min from a single left ventricular epicardial site in patients undergoing coronary artery surgery during cardiopulmonary bypass. To the best of our knowledge, they are the only available MAP data for comparison purposes in human *in-vivo*. Our results are in very close agreement with these data (see Figure 5A).

A very close agreement with experimental data is also found in the tissue simulations. Indeed, the two middle panels of Figure 8A show a direct comparison of the time course of ${\left[K^{+}\right]}_{o}$ in two locations (corresponding to the proximal and distal ends of the BZ–2.0 cm and 2.9 cm, respectively) with experimental data from Coronel et al. (1988) and Hill and Gettes. (1980). The quantitative agreement is, again, very notorious. It should be highlighted that all the mentioned experimental data were not used to train or fit the model, and were not even considered when defining our ischemic cellular and tissue model. The data on potassium accumulation and AP behaviour were only looked at *a posteriori* to test the validity of the model. The good agreement between our results and the experiments demonstrates the goodness of the computational model from a predictive point of view.

As for the time course of the net efflux rate resulting from our simulations, it is also in accordance with experimental measurements in animals (Wilde et al., 1990). In this experiment, a rapid increase in the net efflux rate, followed by a decrease to almost zero and an eventual slower increase, was found in globally ischemic mammalian hearts. The values they obtained for the net efflux rate are in the range of our simulated values.

Unfortunately, to our knowledge, the rise in ${\left[K^{+}\right]}_{o}$ in the acutely-ischemic human heart has not been measured directly, so direct comparisons with our results cannot be made. In 1970, Parker et al. (1970) measured potassium concentration in human and reported an increase during acute ischemia. Unfortunately, they measured potassium concentration in the arterial flow but not in the myocardial interstitium. In 2014, Kazbanov et al. (2014) inferred the time course of ${\left[K^{+}\right]}_{o}$ in the *in-vivo* human ischemic heart during 180 s using a very indirect methodology. Although their results cannot be regarded as proper actual ${\left[K^{+}\right]}_{o}$ measurements, the triphasic trend they reported agrees with the results in animal and with our own simulation results. Also, they report a calculated mean increase in ${\left[K^{+}\right]}_{o}$ of 2.2 mmol/L after 2.5 min, which is in the range of our results (2.57 mmol/L, see Figure 2A).

As for the dispersion of ${\left[K^{+}\right]}_{o}$ during regional ischemia and size of the BZ, our model predicted a BZ of about 1.2 cm at 10 min after occlusion (see Figure 6A). These results are in good agreement with the work by Hill and Gettes. (1980); Coronel et al. (1991) that reported a BZ of approximately 1 cm in acute regional ischemia in isolated pig heart 7 min after occlusion. In addition, our model predicts that ${\left[K^{+}\right]}_{o}$ accumulates outside the BZ, in the normal zone, for a distance of about 2 mm, in close agreement with the results from Coronel et al. (1991).

According to our simulations, the injury current during the negative T wave, corresponding to a potential gradient of almost 60 mV accross the BZ (see Figure 9) was of 1.5 μA/mm^3^ at 3 min post-occlusion and 2 μA/mm^3^ at 4 min post-occlusion. These values are found to be in good agreement with the 2 μA/mm^3^ reported by Janse et al. (1980) in canine hearts at 4 min after LAD occlusion. Further, we found that this current was about eight times smaller than the excitatory current provided by the traveling front. This result is higher than the results from Janse et al. (1980) who reported an excitatory current as twice as large as the injury current. However, they computed the current with electrodes spaced a distance between 1.5 and 4 mm which lead to an underestimation of the excitatory current as demonstrated in Coronel et al. (2000). On the contrary, the intracellular depolarizing current computed by our model at the metabolic border was found to be in good agreement with the experiments from Coronel et al. (2000) on porcine heart, and the simulation results from Potse et al. (2007a).

In summary, the good agreement between experimental evidences and the results obtained with our model, both in the single cell and tissue levels, enables us to suggest new hypothesis regarding the intimate mechanisms of ischemia-induced hyperkalemia, as will be explained in the next sections.

### 4.3 The mechanisms of extracellular potassium accumulation

Experimental evidence shows that extracellular potassium concentration in the interstitium of acutely ischemic myocardial tissue shows a triphasic time pattern, with an initial rapid rise being followed by a plateau phase and a secondary slower increase. Regardless of the intimate mechanisms involved, dynamic changes in ${\left[K^{+}\right]}_{o}$ must be due to an imbalance between potassium efflux and influx to and from cardiomyocytes. If the former is higher than the latter, ${\left[K^{+}\right]}_{o}$ will increase, and *vice versa*. Said triphasic pattern is the result of the interplay between efflux and influx during the acute phase of ischemia.

#### 4.3.1 Primary rising phase

Due to the difficulties related to the direct measurement of the individual contributions to the rapid primary rise of ${\left[K^{+}\right]}_{o}$ , the classical hypotheses that aim an explanation are basically of speculative nature. In summary, the following factors have mainly been hypothesized to play a role: a) a partial inhibition of the NaK pump, b) an enhancement of the *I*_*K*(*ATP*)_ current, c) an increase of sodium influx and d) a reduction of the interstitial volume. Also, the discussion as to whether the extent to which this rise is due to an increased efflux and/or a reduced influx is still not resolved (Wilde and Aksnes, 1995; Weiss et al., 2017).

Regarding the partial inhibition of the NaK pump, due to the decrease in the intracellular ATP/ADP ratio, this effect is included in our model. Indeed, the last row in Figure 4 shows the decrease in magnitude of the current carried by the pump. Moreover, the bars in Figure 3 (and also the results shown in Supplementary Figure S3) show that the magnitude of the influx rate through the pump rapidly decreases during the primary rising phase of ${\left[K^{+}\right]}_{o}$ , with the decrease in the magnitude of the potassium influx rate seen in Figure 2C being the direct consequence of this partial inhibition. Indeed, our results clearly show that this reduction is pivotal in explaining the primary rise in ${\left[K^{+}\right]}_{o}$ , because the reduction in the influx (55% in 2.5 min), almost exclusively generated by the NaK pump depression, is much higher than the increase in the efflux during this phase (10% in 2.5 min). This confirms the experimentally derived hypothesis regarding the importance of the NaK pump inhibition. Also, this is in accordance with the main conclusion of a previous modelling study (Terkildsen et al., 2007). According to their model, the inhibition of the NaK pump is the major driving force for potassium loss. This is difficult to correlate with experimental findings because quantification of the influx rate of the NaK pump relies on indirect methods (Wilde and Aksnes, 1995). Yet, evidence of an ischemia-induced depression of the pump exists (Bersohn et al., 1982; Mitani and Shattock, 1992).

Regarding the enhancement of the ATP-sensitive potassium current, Our results show that, indeed, this factor is not as essential as the depression of the NaK pump in the primary rise of ${\left[K^{+}\right]}_{o}$ . On the one hand, the efflux rate increase related to the enhancement of *I*_*K*(*ATP*)_ 5 min post-occlusion is only 5.8 (*μ*mol/L)/s (see Figure 3D ;Supplementary Figure S3), which is much smaller than the concomitant influx rate decrease (20 (*μ*mol/L)/s). In that same time period, the increase in the efflux rate provoked by the increase in *I*_*K*1_, for instance, is 5.6 (*μ*mol/L)/s, almost equal to the *I*_*K*(*ATP*)_ case. The lack of a higher increase in the *I*_*K*(*ATP*)_-related efflux seems to be paradoxical with the continuous increase in the peak value of the current shown in the third row of Figure 4. The explanation, also pointed out by Wilde and Aksnes. (1995); Weiss et al. (2017), is that the *I*_*K*(*ATP*)_ increase is a self-limiting mechanism in terms of potassium efflux, because the continue increase in its peak is counteracted by the decrease in its duration caused by the continuous reduction in *APD*_90_. Thus, our results show that *I*_*K*(*ATP*)_ does play a role in the primary potassium accumulation phase, but it is quantitatively less important than other currents (mainly the NaK pump).

Regarding the modification in sodium influx and its role in the primary phase of potassium accumulation, the classical hypothesis is that the increase in potassium efflux is a passive consequence of an increased sodium influx to maintain electroneutrality (Wilde and Aksnes, 1995; Shivkumar et al., 1997). Further, there is evidence that ${\left[Na^{+}\right]}_{i}$ increases during acute ischemia (see Wilde and Aksnes (1995) for a review), although the exact extent is unknown. ${\left[Na^{+}\right]}_{i}$ does indeed increase in our simulations (from 7.3 mmol/L in normoxia to 9.3 mmol/L in 5 min, as seen in Supplementary Figure S7). Although we did not analyze the causes of this increase, the partial inhibition of the NaK pump seems to be the main factor responsible. This increase in intracellular sodium is necessarily coupled to the increase in extracellular potassium (Wilde and Aksnes, 1995; Shivkumar et al., 1997). According to our results, however, the change of one of them is not the driving cause of the other, but rather they are both caused by their common underlying mechanism: the reduction of the NaK pump itself. As pointed out in Terkildsen et al. (2007), the partial inhibition of the pump accounts for both concentration changes.

Finally, a shrinkage of the interstitial extracellular space provoked by the ischemia-induced alteration of osmotic pressure has also been hypothesized to play a role in the increase in ${\left[K^{+}\right]}_{o}$ (Wilde and Aksnes, 1995). We preferred not to include this mechanism in our main set of simulations due to its intrinsically different nature compared to the efflux-influx interplay. However, we conducted separate simulations to assess the quantitative importance of the extracellular volume regulation. The main result is shown in Supplementary Figure S8, where we compare the basic “control” simulation of Figure 2A with a situation in which extracellular volume was reduced by 9% in 30 min (data extrapolated from Terkildsen et al. (2007)). The curves show that the effect of extracellular shrinkage was almost negligible during the primary ${\left[K^{+}\right]}_{o}$ rising phase, and its effects were only notorious (but very limited) in the long term. Indeed, the contribution of the extracellular volume reduction was only +0.6 mmol/L after 20 min. These results are quantitatively similar to a previous modeling study (Terkildsen et al., 2007) and experimental results (Yan et al., 1996).

#### 4.3.2 Transition to the potassium plateau

One of the most intriguing and novel result from our simulations refers to the causes of the smooth transition to the extracellular potassium plateau and its relationship with AP alternans. As explained in the Results section, the model predicts the appearance of electrical alternans between the fourth and the sixth minute of ischemia in the isolated cell model (Figures 5B,E). Moreover, the tissue simulations also show AP alternans in the distal altered zone (Figure 8B). This is in correspondence with experimental results in animals. Indeed, these type of alternans, occurring at normal/low stimulation frequencies (1 Hz in our case) around 5 min post-occlusion (or even earlier), are a well-known feature of acute ischemia and have been observed in AP or MAP recordings and/or in electrograms (Kléber et al., 1978; Nakashima et al., 1978; Janse, 1990; Martišienė et al., 2015; Watanabe and Gettes, 2016). Moreover, the experimentally-obtained waveforms of the alternating APs are very similar to our simulated results shown in Figure 5B. The “long” AP has the upstroke divided in two phases, whereas the “short” one lacks the secondary phase. These exact features were found in the APs directly recorded in pig hearts 5 min after clamping the LAD coronary artery in pig hearts [see Figure 2 in Kléber et al. (1978); Figure 4 in Janse (1990)]. The morphology of the AP alternans seen in Figure 1 in Downar et al. (1977) are very similar to those found in our tissue simulations (Figure 8).

In our “0D simulations” (isolated cell), the onset of the ${\left[K^{+}\right]}_{o}$ plateau coincides with the alternans phase (Figure 5E). Similarly, in our “1D simulations” (heterogeneous tissue), the alternating period is also simultaneous with (x = 3.3 cm in Figure 8) or precedes (x = 2.0 cm and x = 2.9 cm in Figure 8) the onset of the plateau. In view of this result, we hypothesize that AP alternans are the main cause of the stabilization in the ${\left[K^{+}\right]}_{o}$ rise. To confirm this hypothesis, we conducted a separate simulation in which the alternans were inhibited by artificially enhancing the *I*_*CaL*_ current at critical instants of the simulation in which the alternans were just about to appear. The results, shown in Supplementary Figure S9, indicate that the absence of AP alternans abolishes the potassium plateau.

According to our simulations, the flattening of the ${\left[K^{+}\right]}_{o}$ curve occurs mainly because of a drastic reduction in potassium efflux rate (see Figure 2C) caused by the AP alternans. Comparing Panels C and D from Figure 3, indicates that this reduction is mostly due to the depression of the efflux carried by *I*_*Kr*_ and, to a lesser extent, by *I*_*Kb*_. These currents are active during the AP plateau (i.e., during systole). When alternans appear, the duration of the plateau is reduced at least in one of every two APs, which provokes a reduction in the potassium carried by said currents. This reduces potassium efflux and decelerates the “macro” ${\left[K^{+}\right]}_{o}$ curve (Figure 5A) due to the behaviour of the “micro” ${\left[K^{+}\right]}_{o}$ fluctuations (Figure 5E).

Different experimental results have been published in which APD and ${\left[K^{+}\right]}_{o}$ (or potassium activity) are measured simultaneously during no-flow acute ischemia (Weiss and Shine, 1982b; Kléber, 1983; Weiss and Shine, 1986; Weiss et al., 1992). In one case (Weiss et al., 1992), the potassium plateau was not reached during the duration of the experiment. In another (Weiss and Shine, 1982b), the existence of electrical alternans cannot be inferred (see their Figure 2). This could be due to the low resolution with which they measure APD (one measurement per minute), which makes it impossible to detect short periods of AP alternation. However, in one publication (Kléber, 1983), APD is measured with enough resolution as to detect alternans. In their Figure 2A, alternans are found between 4.5 and 5.5 min post-occlusion, just as in our case. The alternans occurred before the onset of the extracellular potassium plateau. In our “1D simulations” (heterogeneous tissue), similar results have been found. Moreover, Watanabe and Gettes. (2016) found AP alternans in pig hearts subject to regional ischemia before and during the plateau phase (see their Figure 5).

The mechanisms that give rise to the ${\left[K^{+}\right]}_{o}$ plateau phase are not yet clarified. According to Weiss and Shine. (1986), the reduction of APD as ischemia progresses contributes to the existence of the plateau. They found that the onset of the plateau occurred when APD had reduced to about two-thirds. In our “0D simulations”, that was the case when the curve begins to flatten just when the alternans began to appear: the *APD*_90_ value transitioned from its normoxic value of 289 milliseconds–192 milliseconds just before the onset of alternans, which corresponds to a 66% percentage reduction of the control value. They hypothesized that the decrease in APD reduced the potassium efflux rate until it equaled the influx rate, giving rise to the plateau. This is consistent with our results (Figures 2C, Figure 5).

Indeed, in our “0D simulations”, the net potassium efflux rate is almost zero in the plateau around the 10 min mark. It stays below 2.2 (*μ*mol/L)/s (10% of its peak value) from the 6.8 to the 13.4 min mark. During this period, influx and efflux rates are almost balanced with a value of ≈10 (*μ*mol/L)/s, which is much smaller than in normoxia (≈34.5 (*μ*mol/L)/s). On the one hand, influx rate is smaller than in normoxia because of the progressive partial inhibition of the NaK pump. On the other hand, efflux rate is also smaller due to the short duration of the AP plateau, which practically eliminates all potassium currents except for *I*_*K*1_ and *I*_*K*(*ATP*)_. The former is not AP plateau-dependent (due to its inward rectification nature), while the latter has a high peak due to the low value of the ATP/ADP ratio at this point in time. The overall result is a new stable ${\left[K^{+}\right]}_{o}$ at ≈ 11.7 mmol/L, the value up to which the initial 6.8 min lifted extracellular potassium. This can be better understood when comparing the “micro” ${\left[K^{+}\right]}_{o}$ fluctuations in Panels C and F from Figure 5: while both correspond to stable situations (normoxia in Panel C, potassium plateau in panel F), the smaller fluctuations in the latter case are the consequence of smaller (but approximately equal) efflux and influx compared to the normoxic case.

A further proof of the influence on APD on the plateau phase is given in Supplementary Figure S9. As explained above, artificial interventions were made to eliminate AP alternans. The lack of a plateau phase in this separate simulation is because such interventions did not only abolish alternans but also maintained *APD*_90_ levels high compared to the control simulation.

Another classical explanation of the potassium plateau, different to considerations on APD, is related to the behaviour of the NaK pump. This has been argued both from experimental observations (see Wilde and Aksnes. (1995) for a review) and from previous computational modeling studies (Rodríguez et al., 2002; Terkildsen et al., 2007). Simulations carried out by Terkildsen et al. (2007) showed that the time course of *I*_*NaK*_ is triphasic and, according to them, the ${\left[K^{+}\right]}_{o}$ plateau is a reflection of the plateau in the activity of the NaK pump, which, in turn, is due to the biphasic nature of the time course of intracellular ADP. In our simulations, we also found a deceleration in the decrease of the magnitude of the NaK-related potassium influx (see the bars in Figure 3 an also Supplementary Figure S3) which was concomitant to the potassium plateau, but the elimination of the alternans (Supplementary Figure S9), while maintaining the NaK pump plateau, abolished the potassium plateau. It must be noted that the model used by Terkildsen et al. is different from ours, as it corresponds to the previous generation of AP models (Luo and Rudy, 1994) and is not as comprehensiveness as the model used in this study (O’Hara et al., 2011). Also, their model of ischemia is also less comprehensive. Using a similar AP and ischemia model, Rodríguez et al. (2002) explained the plateau by the combined effect of the above discussed self-limiting effect of the activation of *I*_*K*(*ATP*)_ and an enhancement of the NaK pump which was not found with our model. In the model used by Rodriguez et al., this enhancement was due to the regulatory effect exerted by the increase in both ${\left[K^{+}\right]}_{o}$ and ${\left[Na^{+}\right]}_{i}$ on the pump activity. Although this regulatory effect was included in our formulation of the NaK pump following Cortassa et al. (2006); Terkildsen et al. (2007), its effect was more modest than in the former model. Our model is consistent with the fact that the increase produced by ${\left[K^{+}\right]}_{o}$ elevation between 4 and 12 mmol/L is mild (Gadsby, 1980; Glitsch et al., 1981; Weiss and Shine, 1986) and surpassed by the opposite effect caused by the decrease in the intracellular ATP/ADP ratio.

#### 4.3.3 Secondary rising phase

The prior hypotheses regarding the causes of the secondary slower rise in potassium accumulation are scarce. Classically, this phase has been related to cell irreversible damage (Weiss and Shine, 1982b). It is not completely clear if the cause is related to an increase in potassium efflux provoked by the loss of sarcolemmal integrity or by a further depletion of NaK pump activity (Weiss and Shine, 1986). According to a previous modeling study, the latter could be the main cause (Terkildsen et al., 2007). From our results, a complementary hypothesis may be established. Cell membrane damage was not included in our model, so our results discard the relevance of this factor. According to our simulations, potassium efflux rate almost plateaus during the secondary rise in ${\left[K^{+}\right]}_{o}$ while the magnitude of the influx keeps decreasing, giving rise to a secondary increase in the net potassium efflux rate (see Figure 2C). The fact that the efflux is maintained is mainly due to plateauing of both *I*_*K*1_ and *I*_*K*(*ATP*)_ (see the bars in Figure 3, the ionic currents in Figure 4 and also Supplementary Figure S3). As for the secondary decrease in the influx rate, it is due to the secondary depression of the NaK pump (see the same figures), which is consistent with the fact that intracellular ATP levels keep decreasing, though at a smaller rate compared to the primary phase (Figure 1).

This phenomenon may be alternatively explained from a different perspective. The rate of rise in the secondary ${\left[K^{+}\right]}_{o}$ increase (from ≈14 min onwards, see Figure 2A) is in the same order of magnitude as the primary rise when the cell is quiescent (lower curve in Figure 2B). This can also be seen in experimental results (e.g., Wilde and Aksnes. (1995); Figure 1). The reason is that both situations are very similar in terms of the electrical activity. On the one hand, in the quiescent case, there are no APs, so the efflux and influx completely depends on the diastolic currents. On the other hand, in the secondary rise when pacing, the isolated cell (Figure 4, last row at the 20 min mark) and the ischemic tissue (Figure 8, last row at the 13 min mark) are unresponsive, having lost excitability due to the high ${\left[K^{+}\right]}_{o}$ value.

#### 4.3.4 The potassium border zone

The potassium gradient that is established during acute regional ischemia near the metabolic border, the BZ, is believed to be related with the extracellular potassium transport (Coronel et al., 1988; Coronel et al., 1989). However, our simulations indicate the size of the BZ to be related to the electrotonic coupling between cells close to the metabolic border, with a minor role of the extracellular potassium transport in modulating the BZ (see Figure 6B). Our results indicate that, an effective modulation of the BZ by potassium transport requires a ten-fold increase in the potassium diffusion coefficient (see Figure 6B). These findings are in agreement with the work from Potse et al. (2007b) who conclude that the mechanism that leads to a smooth distribution of ${\left[K^{+}\right]}_{o}$ cannot be physical diffusion alone. They suggest pulsating flow in the arterial and venous bed as possibly responsible for the increase in the potassium diffusion coefficient, a hypothesis also proposed by Janse and Wit. (1989). However, it is retained very unlikely that pulsating flow can increase the effective diffusion coefficient ten times with respect the measured diffusion constant of potassium in water, as to be able to sustain the smooth distribution of extracellular potassium observed in acute ischemia. However, the results in Figures 7B, D, indicate a transport of potassium from the ischemic toward the normal myocardium where the excess of potassium is removed by the washout. Our results clearly evidenced the need of systolic currents i.e., a propagating front in the tissue, for the development of the BZ. In fact, considering only diastolic currents as in the work by Niederer. (2013) leads to a BZ of only few millimeters wide and a limited rise of the extracellular potassium. Considering a BZ equally ischemic, the mechanism revealed by our model says that electrotonic coupling modifies the net potassium efflux in the BZ by modulating the current through ionic channels responsible for potassium efflux, with cellular potassium loss being similar in closely adjacent cells. This explains the small invasion of ischemic conditions within the healthy metabolic area, limited by the presence of potassium washout in the unaltered tissue. Moving into the ischemic zone, the effect of the metabolic border reduces until reaching a situation like what is seen in an isolated cell. In this regard, enhancing potassium diffusion makes this transition smoother as shown in Figure 6B.

### 4.4 Limitations of the study

Our study presents several potential limitations. The first one is that the simulation of progressive acute ischemia relies on the chosen time courses of the ischemic parameters which are imposed to the model (see Figures 1A–D). They have been taken from experimental measurements or calculations reported in the literature (Weiss et al., 1992; Daleau, 1999; Sakamoto et al., 2000; Terkildsen et al., 2007; Gautier et al., 2008). None of them correspond to measurements in human hearts (due to the lack of data) but rather belong to different species (guinea-pig and rabbit) and were obtained in different laboratories and contexts. However, our additional simulations shown in Supplementary Figures S1, S3, as already discussed, indicate that the results obtained with different ischemic parameter time courses are qualitatively similar, so the main conclusions of our study are not hampered by this potential limitation.

Another potential limitation is related to the fact that our results have been obtained with a particular AP model (O’Hara et al. (2011), so the results may be model-dependent. We preferred to use the model by O’Hara et al. because it has been widely used in the past decade and it was adopted by an expert group of the Federal Drug administration (FDA) as the starting point for developing an *in silico* model suitable for regulatory decision making (Colatsky et al., 2016). However, to asses the impact on the chosen model on the results, we carried out another separate simulation using the more recent human ventricular cell AP model by Tomek et al. (2019). The result is shown in Supplementary Figure S10. Again, the results with both models are very similar and our main conclusions regarding the potassium triphasic time course and the mechanisms responsible for the plateau are still valid.

Our tissue simulations have been performed with a 1D model, which may represent a limitation since the effect of electrotonic coupling is enhanced with spatial dimensionality. However, if the stimulation consists in a planar wave in 2D or 3D this effect is greatly reduced and differences in the results between 1D, 2D or 3D simulations are small. In this regard, our future work will simulate 30 min of dynamic acute ischemia in a real 3D geometry of a biventricular heart considering electrophysiological variability and tissue structure. We hope, in this case to reproduce a realistic border zone, but most importantly, this new set of simulations may provide further insights about the importance of electrotonic coupling in ${\left[K^{+}\right]}_{o}$ accumulation during acute regional ischemia.

One of the main limitations of the study is that our model does not consider the mechano-sensitivity of K (ATP) channels (Van Wagoner, 1993; Kohl et al., 2006; Peyronnet et al., 2016; Quinn and Kohl, 2021). Taking into account that stretch modulates the channel sensitivity to ATP and ADP, this could lead to further shortening of the AP duration and in turn this could affect the results regarding extracellular potassium accumulation and the formation of the BZ. Some models in the literature have considered this effect [e.g., (Li et al., 2006)] by simply scaling the maximum conductance of K (ATP) channels to account for stretch. We chose not to do so due to the lack of data regarding the stretch levels in the CIZ and the BZ. However, we acknowledge that this could affect the main results of this work. For instance, our main findings 1 and 2 could be hampered by the lack of mechano-electric feedback in the model. Further simulations with a model that includes the mechano-sensitivity of K (ATP) channels, as well as other stretch-activated non-specific channels, would be needed to asses its impact on the results, which could be related to possible depolarizing effects in the BZ that could eventually lead to triggered activity. However, such simulations were beyond the scope of the present work.

Moreover, the cellular ischemic model lacks a description of the effect of other ischemia-related parameters, such as cathecolamines, adenosine, fatty acid metabolites, etc., which could affect the outcome of the model. Also, the tissue model was one-dimensional and represented an electrophysiology homogeneous (though metabolically heterogeneous) cardiac strand. While it was useful to study the effects of regional ischemia on extracellular potassium accumulation, it lacks a proper anatomical and structural description of realistic three-dimensional ventricles. In particular, transmural and apex-to-base differences in APs, as well as anisotropy, could not be included in the model. Finally, inter-subject variability was not considered in our study. To do so, a set of population models should be carried out in which some relevant parameters (e.g., maximum conductances of ionic currents) were randomly varied within predetermined ranges. Our results thus correspond to an “average” cell and tissue.

## 5 Conclusion

A model of cardiac tissue electrophysiology to investigate the spatio-temporal evolution of ${\left[K^{+}\right]}_{o}$ across the ischemic BZ was developed. Our main results suggest that ischemia-related extracellular potassium accumulation obeys to intrinsic features of the isolated ischemic cell. The characteristic triphasic time course of ${\left[K^{+}\right]}_{o}$ is related to changes in the balance between the potassium influx and efflux. In this regard, the initial rising phase of ${\left[K^{+}\right]}_{o}$ is mainly caused by a progressive reduction of potassium influx concomitant with a lesser increase in the efflux, whereas the plateau phase is mainly initiated by the appearance of electrical AP alternans provoked by a reduction in excitability together with an increase in *I*_*K*(*ATP*)_ due to the reduction in the intracellular ATP/ADP ratio. Finally, the secondary ${\left[K^{+}\right]}_{o}$ rising phase is associated with an increment of the potassium efflux due to enhancement of *I*_*K*1_ and *I*_*K*(*ATP*)_ and a reduction of the NaK pump activity. Modulation of these mechanisms by electrotonic coupling is responsible for the spatial distribution of ${\left[K^{+}\right]}_{o}$ and the formation of the ischemic BZ, with extracellular potassium transport having a small influence. However, ${\left[K^{+}\right]}_{o}$ transport plays a major role in the stabilization of ${\left[K^{+}\right]}_{o}$ within the CIZ when the tissue becomes non-excitable. The injury current associated with the deep negative T-wave in the ischemic zone is found to be insufficient to depolarize healthy cells in the normal zone. In addition, part of the outward injury current occur inside the altered tissue where the cells are still within the effective refractory period.

## Data Availability

The original contributions presented in the study are included in the article/Supplementary Material, further inquiries can be directed to the corresponding author. The MATLAB and C codes used in the simulations are available upon request to the corresponding author.
