# Supplementary material for: The mechanisms of potassium loss in acute myocardial ischemia: New insights from computational simulations
Source: Front Physiol. 2023 Feb 27;14:1074160. doi: 10.3389/fphys.2023.1074160 (PMC10009276; doi:10.3389/fphys.2023.1074160)
Supplement: Supplementary file 2 [file DataSheet1.docx]

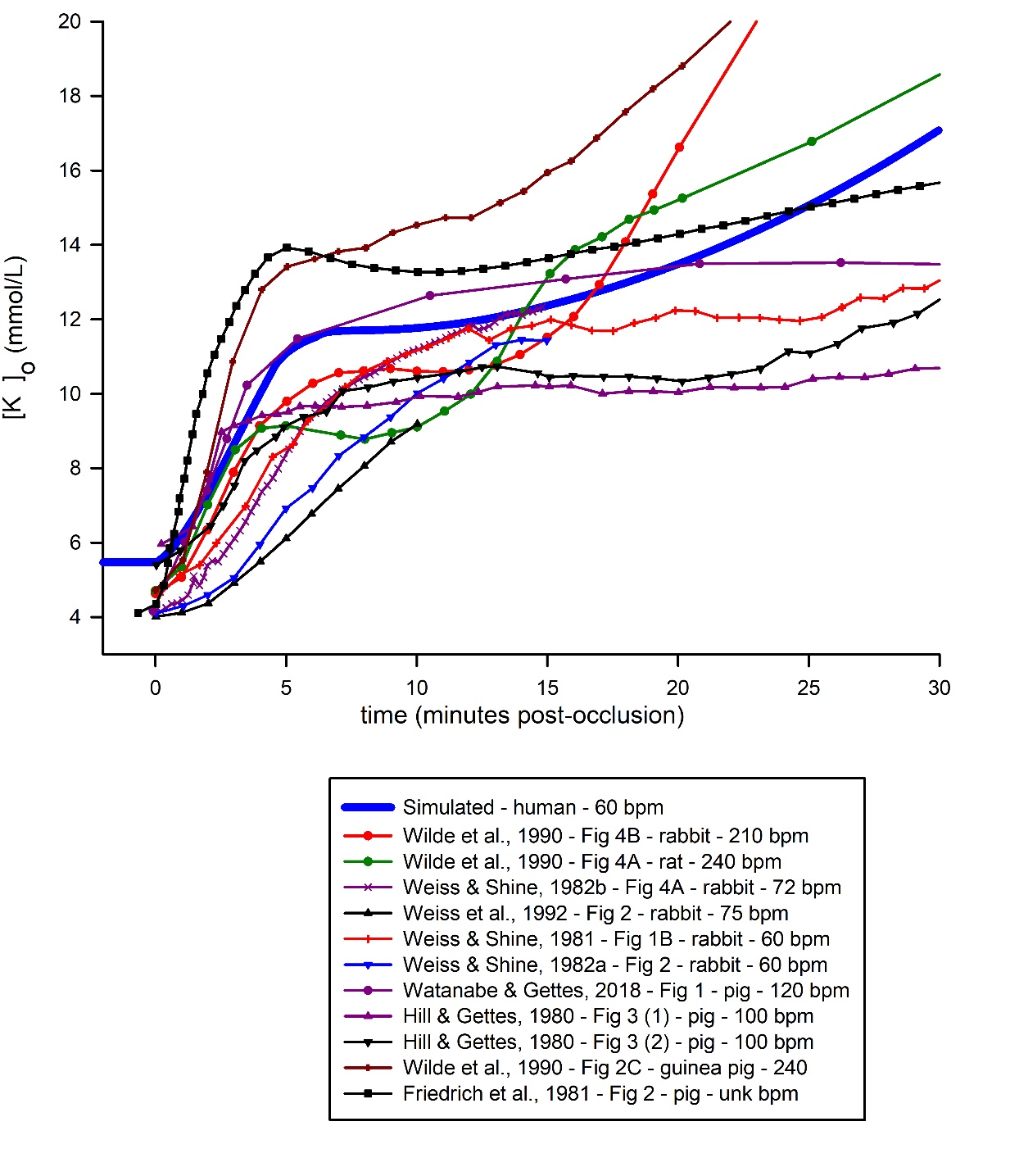
Fig. S1

Comparison between the time courses of extracellular potassium concentration in acute myocardial ischemia. The solid thick blue curve corresponds to our simulation (Fig. 2A of the main text), while the other curves correspond to different experiments (described in the legend, showing paper – figure within the paper, animal species and heart rate).


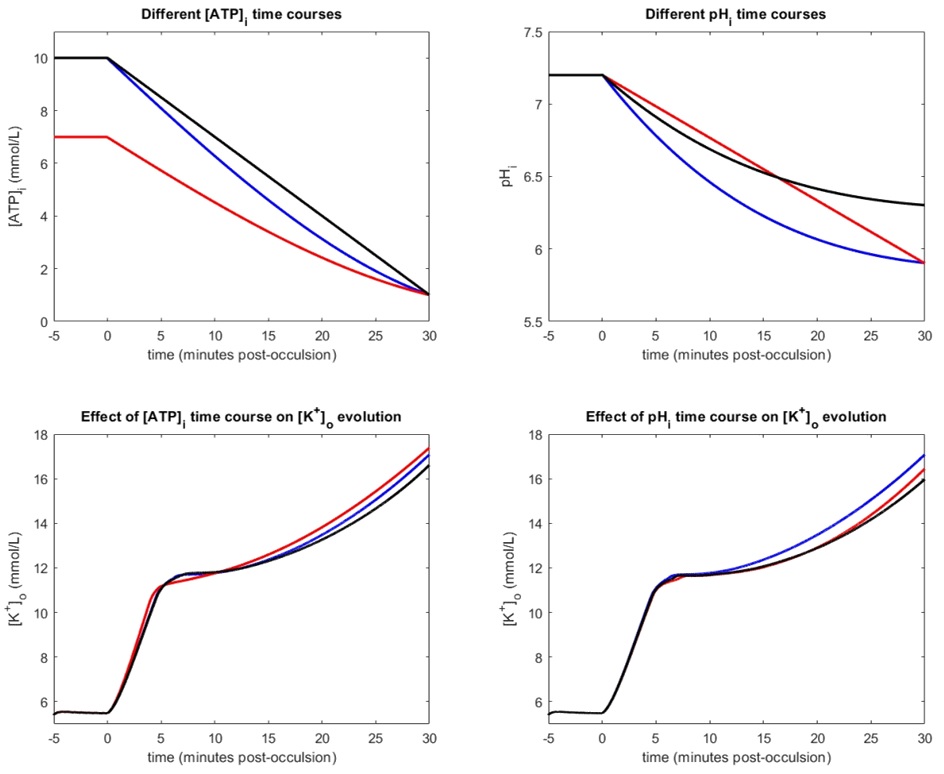
Fig. S2

Left panels: influence of the time course of [ATP]_i_ on extracellular potassium accumulation. The [K^+^]_o_ traces in the lower panel correspond to the same colored [ATP]_i_ plots in the upper panel. The time courses of other ischemic parameters were unchanged in relation to our main simulations. Blue traces: [ATP]_i_ time course from Sakamoto et al. (2000) with a normoxic [ATP]_i_ value as in Cao et al. (2018) and O’Hara et al. (2011) (which corresponds to our main simulations). Red traces: [ATP]_i_ time course from Sakamoto et al. (2000) with normoxic [ATP]_i_ value as in Terkildsen et al. (2007). Black traces: linear [ATP]_i_ time course with normoxic [ATP]_i_ value as in Cao et al. (2018) and O’Hara et al. (2011).

Right panels: influence of the time courses of pH_i_ and pH_o_ on extracellular potassium accumulation. The [K^+^]_o_ traces in the lower panel correspond to the same colored pH_i_ plots in the upper panel. pH_o_ followed a time course parallel to pH_i_ except for a +0.2 units shift. The time courses of other ischemic parameters were unchanged in relation to our main simulations. Blue traces: pH_i_ and pH_o_ (not shown) time course from Sakamoto et al. (2000) with a final pH_i_ value of 5.9 (corresponds to our main simulations). Black traces: pH_i_ and pH_o_ (not shown) time course from Sakamoto et al. (2000) with a final pH_i_ value of 6.3. Red traces: linear time course for pH_i_ and pH_o_ (not shown) with a final pH_i_ value of 6.3.


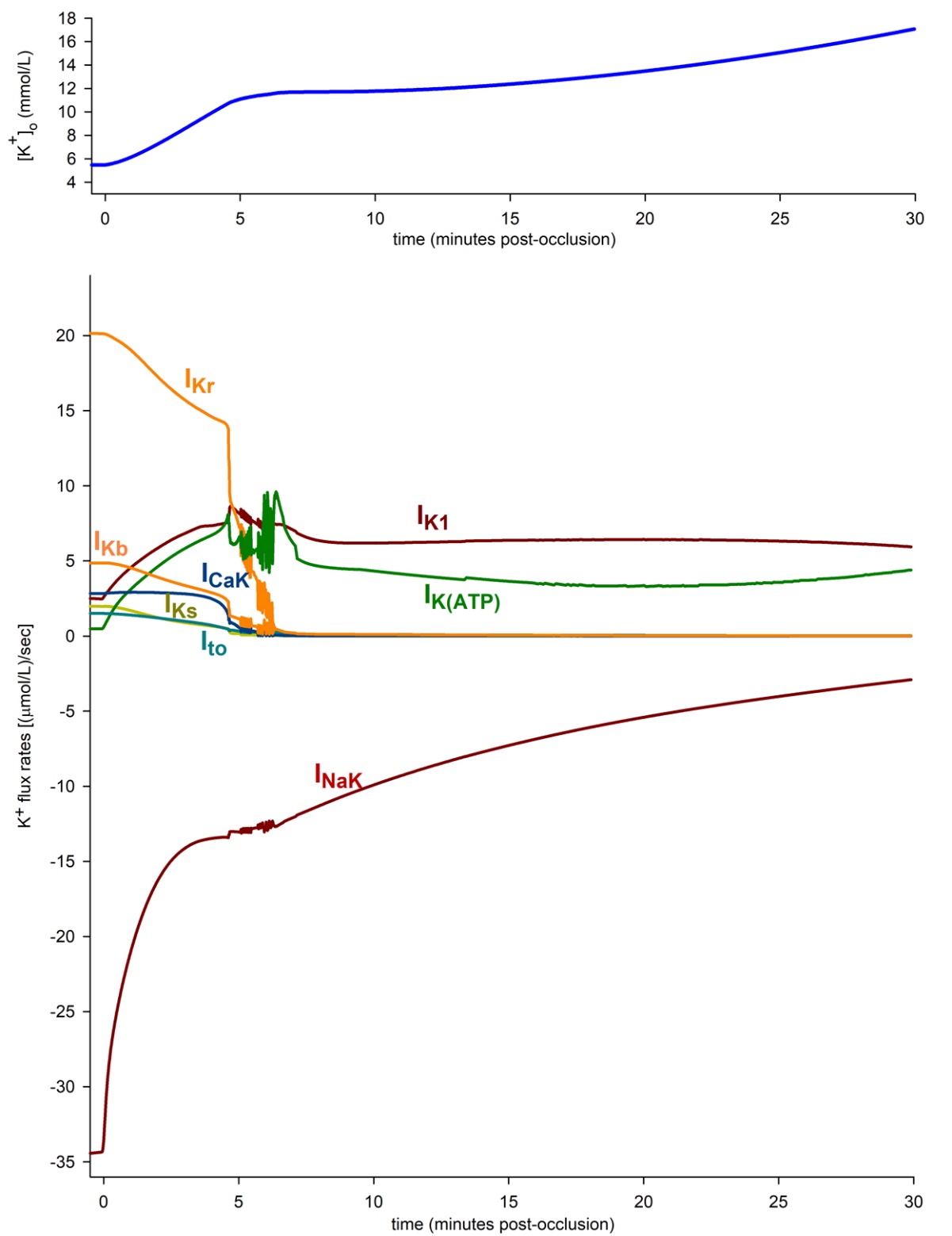


Fig. S3

Time-course of the flux rates corresponding to the individual contributions of all the potassium currents.

**
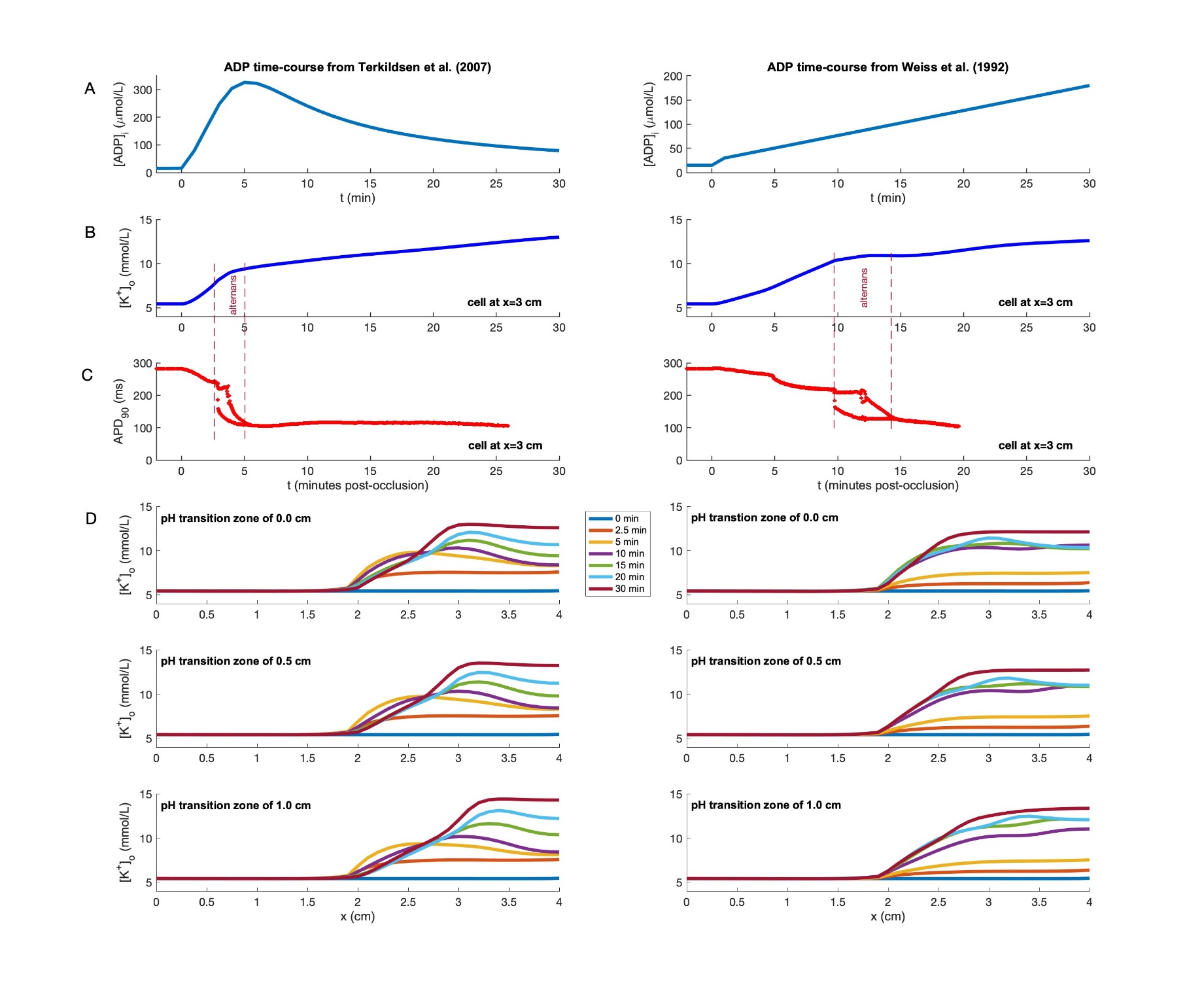
Fig. S4.**

Influence of the time course of [ADP]_i_ on extracellular potassium accumulation. Left column corresponds to ADP data from Terkildsen et al. (2007) (panel A left, our “control” ADP time evolution), while the right column corresponds to ADP data from Weiss et al. (1992) (panel A right, an alternative time course of ADP). Panels B show the time course of [K^+^]_o_ at cell 30 (ischemic zone) in both cases, and panels C depict the correspondent time evolutions of ADP_90_. It can be noted that the ADP data from Weiss et al. (1992) leads to a lower rate of rise of [K^+^]_o_ during the primary rising phase, which delays the potassium plateau with respect to our control simulations based on the data from Terkildsen et al. (2007). However, the results are qualitatively similar. Indeed, a plateau is reached when AP alternans flatten the curve, the same as in our main simulations. Also, the plateau is related to a low value of APD_90_ in both cases. Panels D show the effect of the length of the pH transition zone and the ADP time course in the formation of the [K^+^]_o_ border zone. In can be noted that the qualitative features of the potassium profiles are similar in all cases.


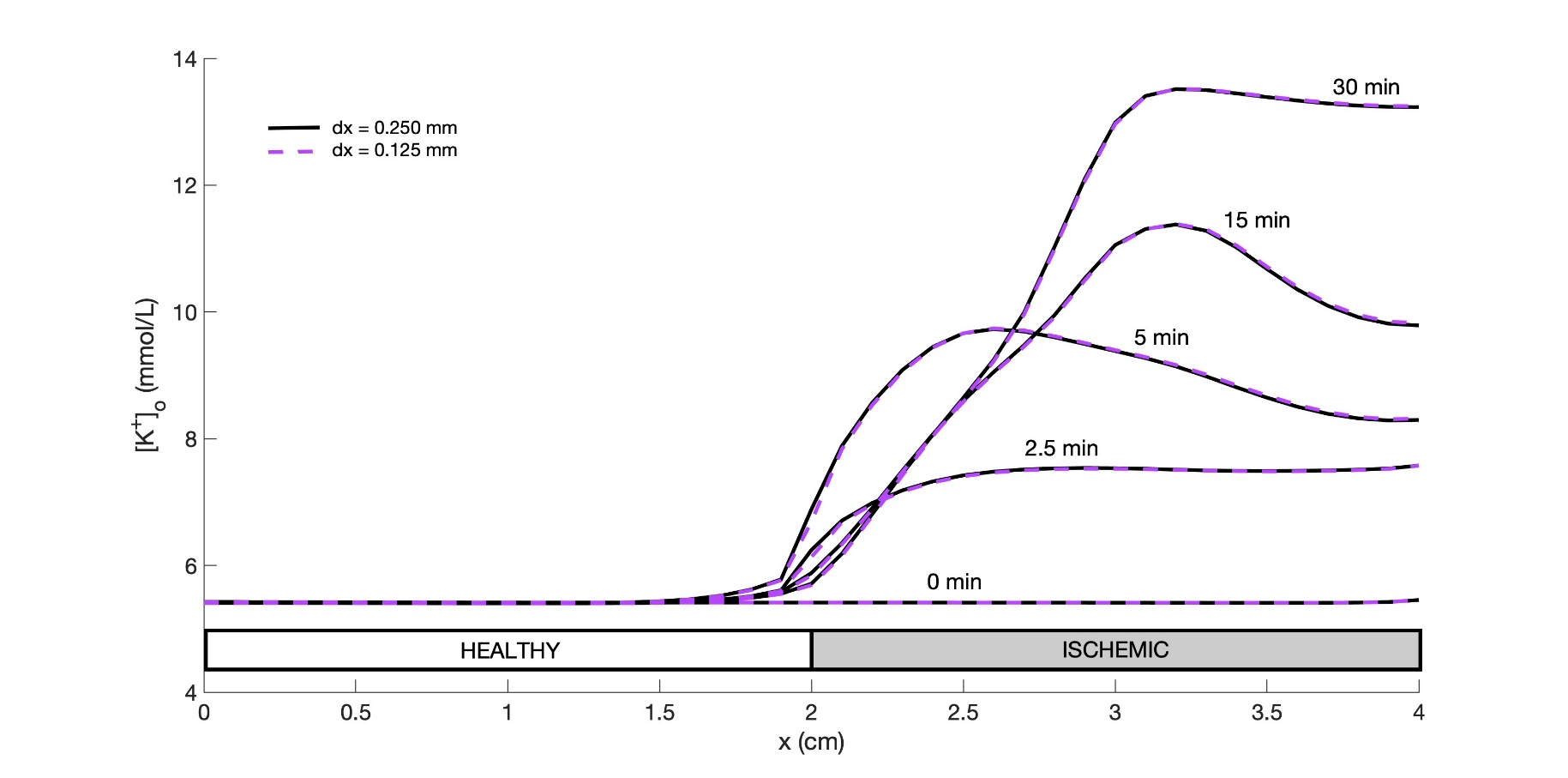


Fig. S5

Effect of space discretization on the extracellular potassium spatial profile at different minutes post-occlusion.


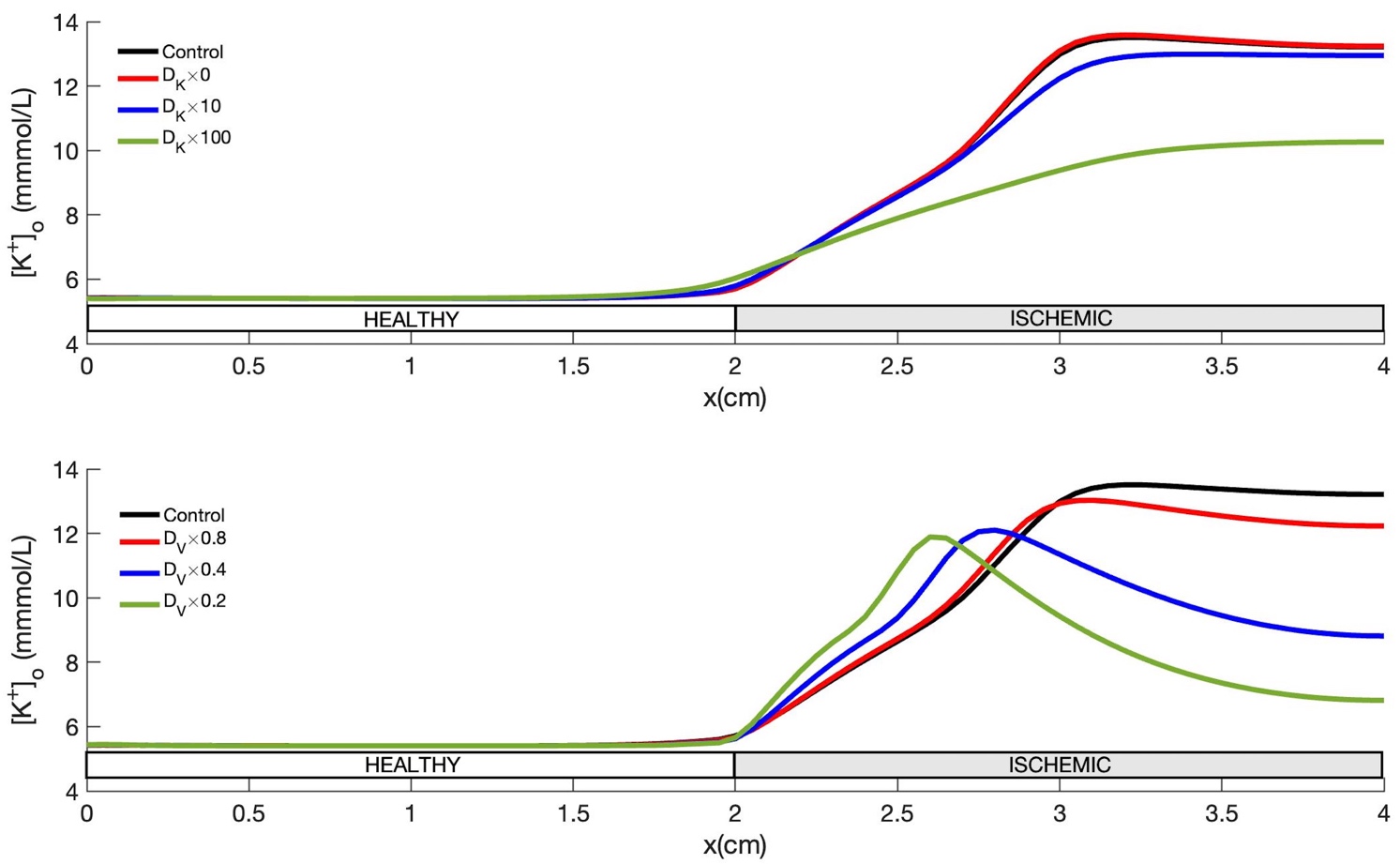


Fig. S6

Effect of the extracellular K^+^ diffusion coefficient (top panel) and tissue conductivity (bottom panel) on the extracellular K^+^ spatial profile 30 minutes post-occlusion.


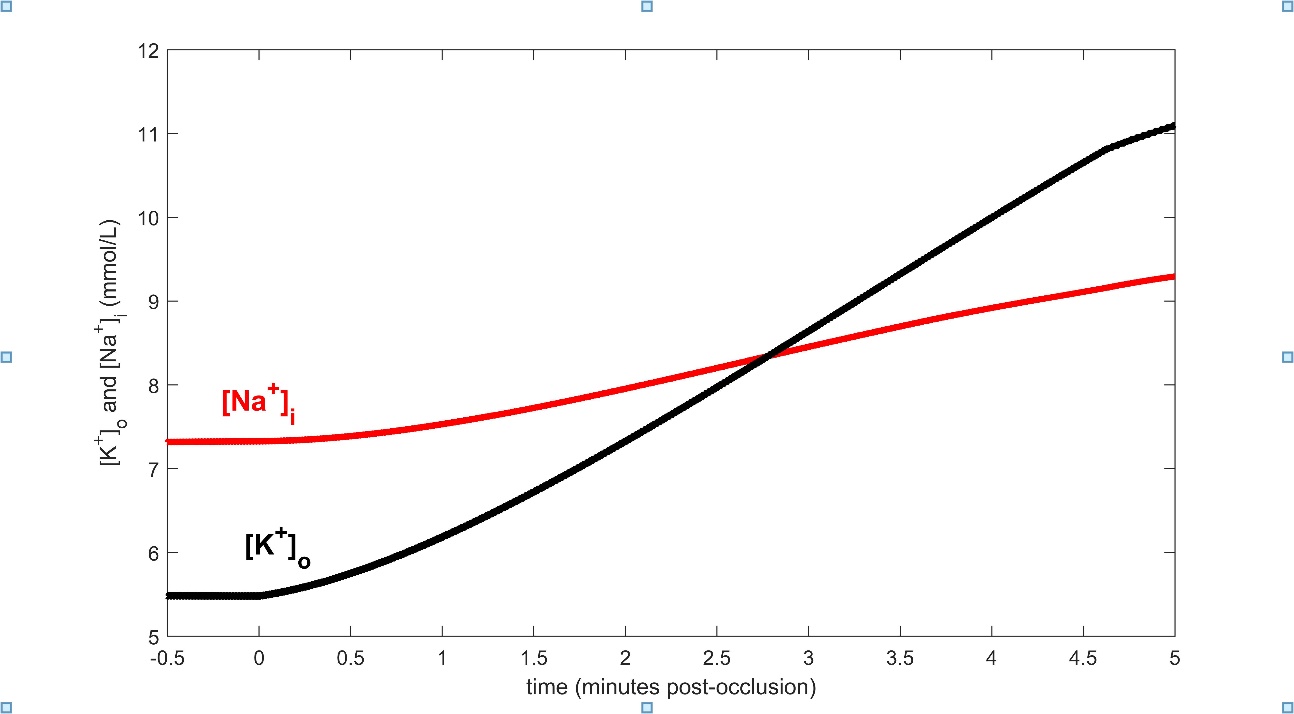


Fig. S7

Increase in intracellular Na^+^ concentration in parallel with extracellular K^+^ concentration in the isolated cell simulation.


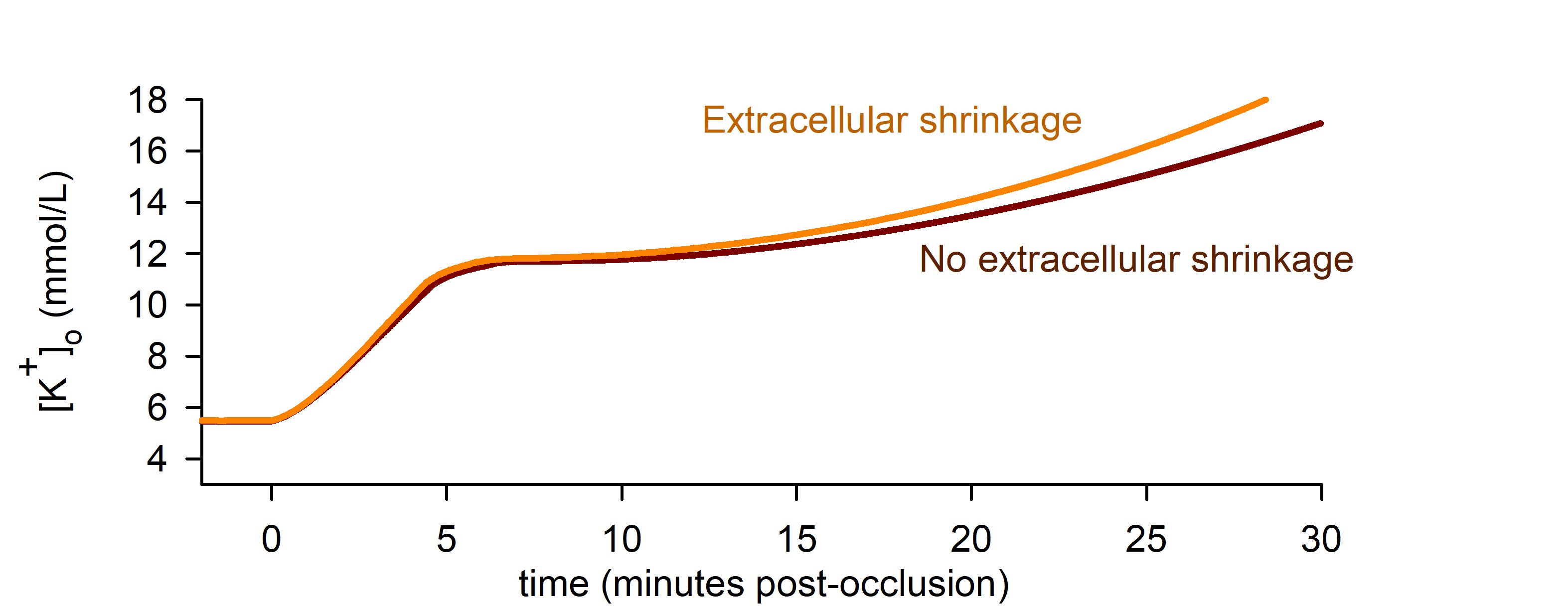


Fig. S8

Influence of extracellular volume on extracellular potassium accumulation. Curve in orange is obtained by imposing an extracellular volume shrinkage of 9% in 30 minutes. See main text for details.


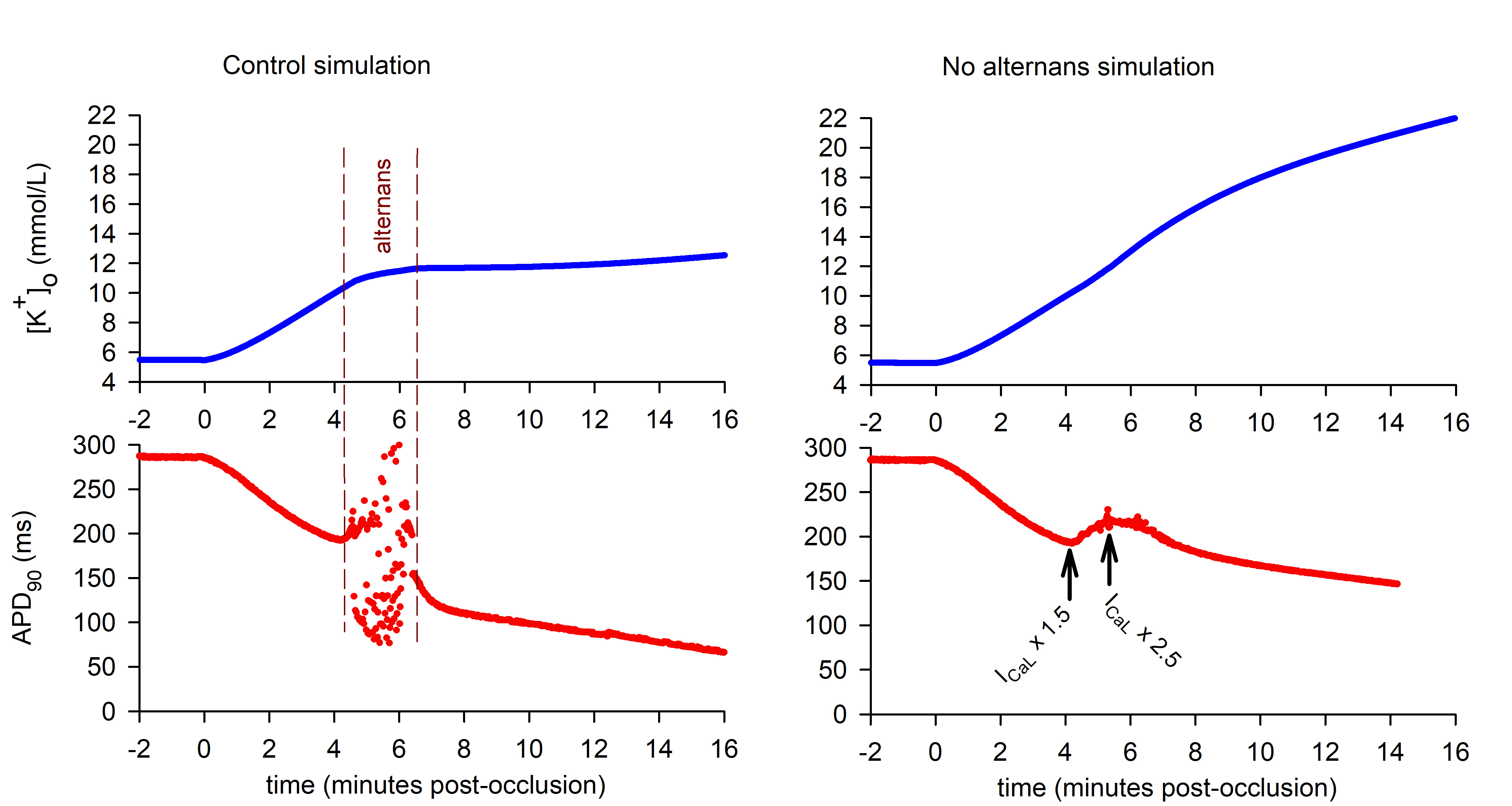


Fig. S9

Effect of AP alternans in extracellular potassium accumulation. The right column shows that the artificial inhibition of AP alternants (see main text) prevents the formation of the potassium plateau phase.


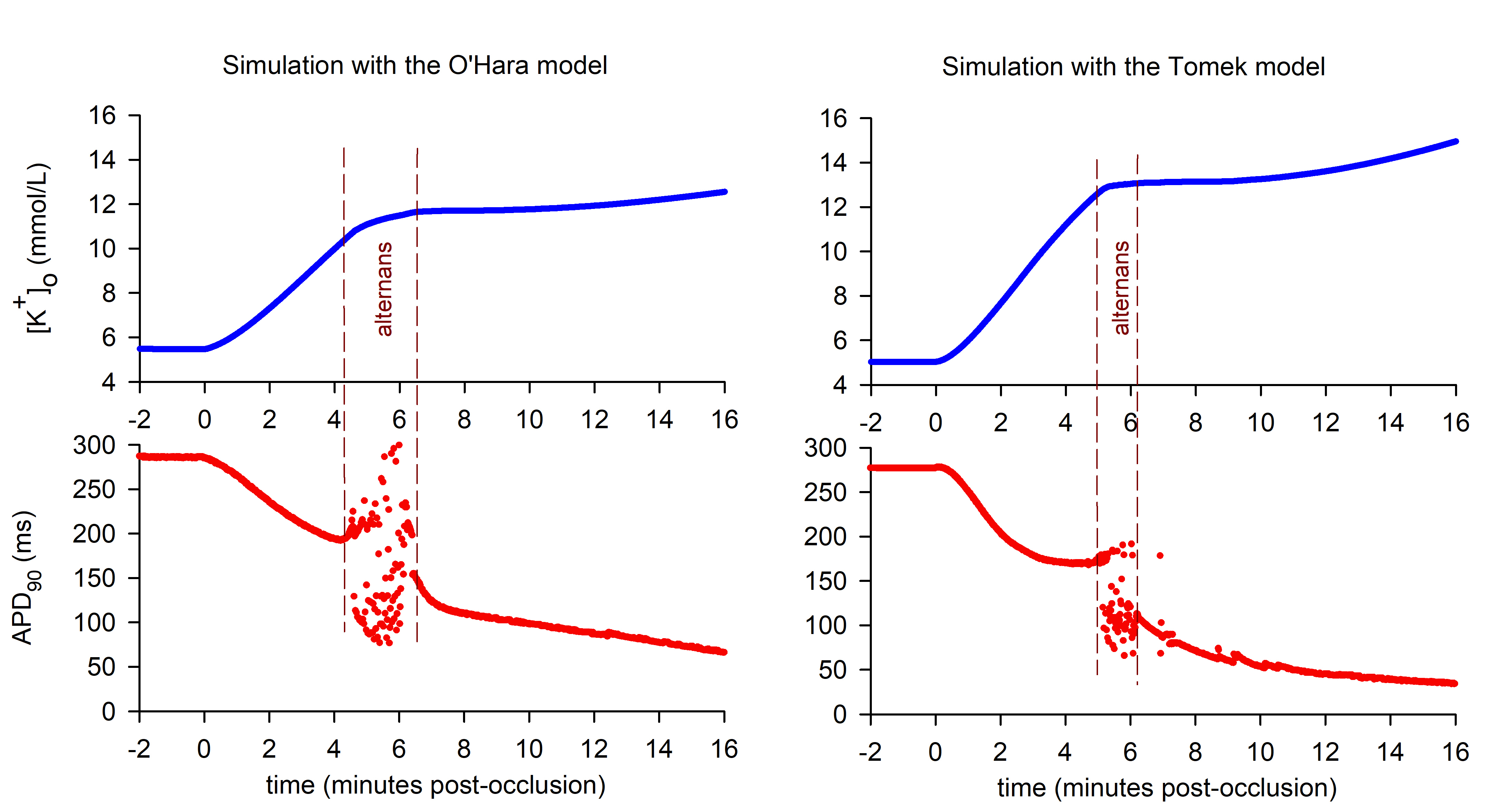


Fig. S10

Effect of different action potential models on extracellular potassium accumulation. Results obtained with the O’Hara et al. (2011) action potential model, used in this work, were compared with those obtained with the more recent Tomek et al. (2019) model. The results obtained with both models are qualitatively similar and the main conclusions regarding the potassium triphasic time-course and the mechanisms responsible for the plateau are still valid.

(see separate file ‘video_dynamic staptiotemporal Ko.avi’)

Movie SM1.

Evolution of the [K^+^]_o_ spatial profile during the 30 minutes after the onset of ischemia (middle panel. The top panel shows the evolution of the electrograms at four different locations within the cable, whereas the bottom panel shows the evolution of the transmembrane action potential at the same locations.
